# A universal cis*-*proline lock defines catalysis in thioredoxin-fold enzymes

**DOI:** 10.1038/s42003-026-10010-8

**Published:** 2026-04-14

**Authors:** Taylor Cunliffe, Geqing Wang, Stephanie Penning, Pramod Subedi, Makrina Totsika, Jason J. Paxman, Begoña Heras

**Affiliations:** 1 Department of Biochemistry and Chemistry, School of Agriculture, Biomedicine and Environment, La Trobe Institute for Molecular Science, La Trobe University, Melbourne, VIC Australia; 2 Centre for Immunology and Infection Control, School of Biomedical Sciences, Faculty of Health, Queensland University of Technology, Brisbane, QLD Australia

**Keywords:** Enzyme mechanisms, X-ray crystallography

## Abstract

Thioredoxin-fold oxidoreductases drive oxidative protein folding and redox homeostasis across all domains of life. They catalyse thiol–disulfide exchange in diverse substrates, yet how they reconcile catalytic precision with substrate diversity remains unclear. Here we show, using high-resolution structures and functional analyses of the *Escherichia coli* oxidoreductase DsbA, that a conserved *cis*-proline loop adjacent to the catalytic Cys–Pro–His–Cys motif serves as a universal catalytic lock. The loop positions the substrate cysteine in a right-handed disulfide geometry optimal for exchange, while surrounding surfaces accommodate sequence variation. Substitution of the *cis*-proline abolishes turnover, whereas mutation of the preceding glycine preserves geometry but reduces efficiency. Comparative structural analyses demonstrate that this *cis*-proline–dependent hydrogen-bonding scaffold is conserved across thioredoxins, protein disulfide isomerases, peroxiredoxins and bacterial Dsb proteins. This conserved mechanism explains how catalytic fidelity is maintained while enabling substrate versatility and provides a foundation for enzyme engineering and therapeutic development.

## Abstract (web summary)

Conserved *cis*-proline loop in thioredoxin-fold enzymes acts as a universal catalytic lock, enforcing optimal disulfide geometry for rapid thiol–disulfide exchange while enabling substrate diversity across oxidoreductases in all domains of life.

## Introduction

Thioredoxin (TRX)-fold oxidoreductases are among the most widespread enzyme superfamilies, catalysing oxidative protein folding and redox regulation across all domains of life. Despite a conserved core fold, lineage-specific insertions, duplications, and oligomerisation have diversified their functions, enabling roles in redox signalling, stress responses, and protein homeostasis^1,2^.

In eukaryotes, TRX-like enzymes, including thioredoxins and protein disulfide isomerases, support protein quality control and are pursued as therapeutic targets in cancer, neurodegeneration, and cardiovascular disease^3–8^. In bacteria, Dsb proteins drive folding of virulence factors and resistance enzymes, making them attractive antimicrobial targets and valuable tools in biotechnology^9–14^. These enzymes catalyse thiol–disulfide exchange with exceptional speed and versatility^1,2^. Yet how they reconcile catalytic precision with broad substrate scope—the precision-versus-diversity paradox—remains unresolved, in part because key enzyme–substrate intermediates are millisecond-lived and rarely captured at atomic resolution^15,16^.

**Fig. 1:**
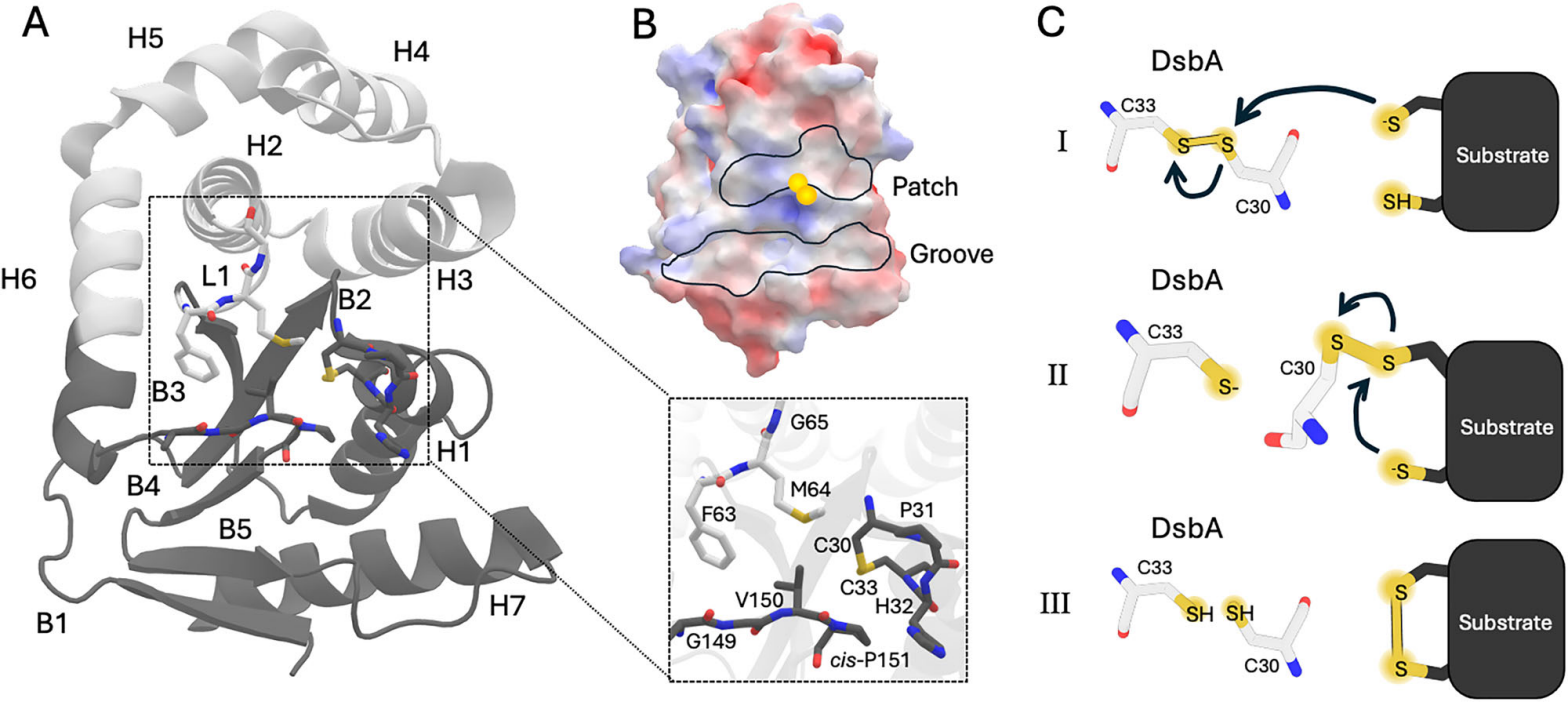
Structural features and mode of action of *E. coli* DsbA. **A** Domain architecture of DsbA (PDB: 1FVK^26^), showing the thioredoxin (TRX)-like domain (β2α1β3–β4β5α7, dark grey), inserted helical domain (α2–α6, white), catalytic C30–P31–H32–C33 motif, the *cis-*proline loop (R148–G149–V150–*cis*P151), and nearby Loop 1 residues (F63–M64–G65)^25,26^. **B** Catalytic surface highlighting the active-site cysteines (yellow spheres), the hydrophobic groove used in partner^41,67,68^/substrate^18,19^ recognition, and the hydrophobic patch engaged in the DsbA-SigA complex^40^. **C** Chemical mechanism of DsbA-catalysed substrate oxidation. (I) Oxidised DsbA, containing a disulfide bond between catalytic cysteines C30 and C33, encounters a reduced substrate protein. (II) A substrate cysteine thiolate nucleophilically attacks Cys30, cleaving the DsbA active-site disulfide and forming a transient mixed-disulfide intermediate, while Cys33 becomes a free thiol. (III) Resolution of the intermediate by a second substrate cysteine results in the formation of an intramolecular disulfide bond in the substrate and leaves DsbA in its reduced state.

We focused on *Escherichia coli* DsbA, the primary disulfide oxidase in Gram-negative bacteria and a prototypical TRX-fold enzyme consisting of a TRX domain with an inserted helical domain (Fig. 1A)^17^. This highly efficient enzyme^18–20^ introduces disulfide bonds into a wide variety of proteins^21^, including toxins such as the heat-stable enterotoxin II^22^, adhesins and pilus-associated proteins like PapD^23,24^, outer-membrane proteins involved in lipopolysaccharide assembly such as LptD^12^, and enzymes contributing to antimicrobial resistance, including β-lactamases^10^. DsbA catalytic activity is mediated by a C–P–H–C redox motif and an adjacent *cis-*proline loop, hallmark features of TRX-fold oxidoreductases (Fig. 1A, B)^25–30^. The chemical mechanism of DsbA-mediated disulfide exchange is well established. In its oxidised state, DsbA presents an active-site Cys30–Cys33 disulfide that functions as the immediate disulfide donor to substrate proteins (Fig. 1C). Catalysis proceeds via nucleophilic substitution, in which a reduced substrate cysteine attacks Cys30, cleaving the DsbA active-site disulfide and forming a transient mixed-disulfide intermediate. Resolution by a second substrate cysteine yields an intramolecular disulfide in the folding protein and leaves DsbA reduced. Cys30 is the cysteine that directly engages the substrate and is intrinsically highly reactive, whereas Cys33 serves as the partner cysteine of the active-site disulfide, contributing to efficient resolution and recycling of the catalytic motif^31–37^. Despite this well-defined chemical mechanism, the structural basis by which DsbA, and TRX-fold enzymes more broadly, accommodate and efficiently catalyse thiol–disulfide exchange reactions in diverse substrates remains unknown.

Here, we address this knowledge gap by defining the molecular mechanism through which TRX-fold enzymes reconcile catalytic precision with breadth. Using DsbA as a model, we captured high-resolution structures of substrate-bound complexes and combined them with mutagenesis, structural analysis, and kinetics. Our results reveal that a conserved *cis*-proline lock adjacent to the catalytic motif acts as a rigid scaffold that enforces the geometry required for thiol–disulfide exchange, while surrounding flexibility permits engagement of diverse sequences. Structural comparisons show that this anchoring geometry is conserved across thioredoxins, protein disulfide isomerases, peroxiredoxins, and bacterial Dsb proteins, establishing a unifying catalytic principle of the TRX-fold superfamily. These findings explain how TRX-fold enzymes reconcile fidelity with promiscuity, account for the evolutionary success of the fold, and provide a framework for engineering oxidoreductases and targeting TRX-like enzymes in therapeutic development.

## Results

### Structural snapshots of DsbA–substrate complexes reveal alternative binding modes

To understand how thioredoxin-fold enzymes engage their substrates, we solved crystal structures of *E. coli* DsbA covalently linked to a peptide from the essential outer-membrane protein LptD^9,12,38,39^. Because DsbA rapidly resolves enzyme–substrate disulfide intermediates during catalysis, we stabilised this transient species by substituting the C-terminal active-site cysteine (Cys33) with alanine. The resulting C33A variant permits formation of the mixed disulfide via Cys30 but prevents its resolution, enabling isolation of a covalent DsbA–substrate complex for structural analysis. This strategy differs from that used previously^40^, which employed an irreversible thioether linkage to trap a substrate mimic rather than the native mixed-disulfide intermediate.

**Fig. 2:**
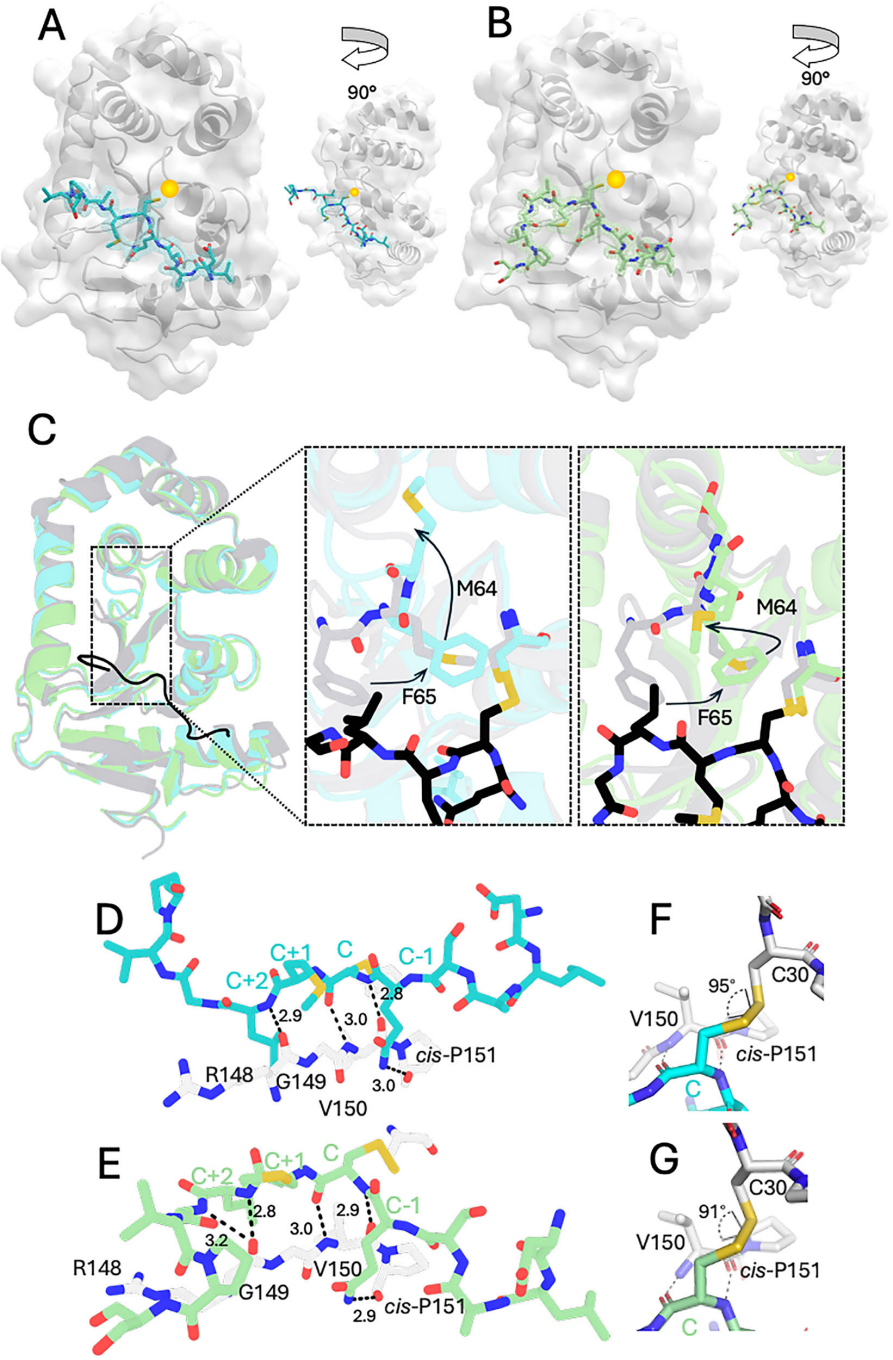
Substrate-binding modes of *E. coli* DsbA. **A**, **B** Crystal structures of DsbA covalently linked to an LptD-derived peptide reveal two binding modes: mode I (cyan) and mode II (green), with 2mFo-DFc map around the bound peptide contoured at 1.0 σ. Peptide cysteine is covalently linked to Cys30. **C** Overlays of apo DsbA (white) with DsbA-LptD binding mode I (blue) and II (green), show Loop 1 flexibility (inset, F63/M64), which enlarges the active-site cavity to accommodate the substrate side chains, without altering the global fold (RMSD 0.51–0.86 Å across 169 Cα atoms). The extent of movement differed between crystal forms. The bound LptD-derived peptide is shown in black. **D**, **E** In both binding modes, the *cis-*proline loop anchors the substrate cysteine through two backbone hydrogen bonds from V150 (V150 C=O···H–N(Cys) (3.0 Å); N–H···O=C(Cys) (2.8 Å)), and an additional bond from R148 (to Cys+2). Extra contacts from P151 (to Cys-1) and, in mode II, from R148 (to Cys+3) further stabilise the complex. **F**, **G** Active-site close-ups for LptD binding modes; *H* mode I (cyan), I mode II (green). In both, the V150–*cis*P151 backbone clamps the substrate cysteine with Cys30 to form a right-handed mixed disulfide (χ₃ ≈ +90°) optimal for SN2-like thiol–disulfide exchange.

The resulting structures, determined at 1.47 Å resolution (Table 1), captured two distinct substrate-binding modes (Fig. 2A*,* B). In both, the substrate cysteine was covalently attached to the catalytic Cys30 of DsbA, and the peptide’s N-terminus extended into the hydrophobic groove of the thioredoxin domain. This groove lies adjacent to the active site (Fig. 1B) and has been implicated in both substrate recognition^21^ and DsbB interactions required for DsbA reoxidation^41,42^. However, in these crystal forms, the peptide backbone adopted different orientations relative to the active site: binding mode I positioned the peptide with its C terminus solvent exposed (Fig. 2A), while mode II aligned the peptide against the catalytic face (Fig. 2B).

**Table 1:** Crystallographic data and refinement statistics

**PDB code**	9Y0O	9Y0N	9Y0M	9Y0P	9Y0Q
**Construct**	G149T	G149K	P151T	DsbA-LptD Complex I	DsbA-LptD Complex II
**Data collection**					
Resolution range (Å)*	43.98–1.79 (1.83–1.79)	43.98–2.06 (2.07–2.00)	44.95–2.88 (3.06–2.88)	43.82–1.47 (1.49–1.47)	48.46–1.47 (1.50–1.47)
Wavelength (Å)	0.95373	0.95373	0.95373	0.95372	0.95372
Space group	*P*2_1_2_1_2_1_	*P*2_1_2_1_2_1_	*P*2_1_	*P*2_1_2_1_2_1_	*P*2_1_2_1_2_1_
Unit cell parameters (Å, °)	68.71, 79.54, 82.43 90, 90, 90	68.89, 79.30, 82.43 90, 90, 90	63.76, 79.75, 103.89 90, 90.19, 90	68.21, 79.33, 82.50 90, 90, 90	31.14, 57.16, 96.91 90, 90, 90
Number of molecules per asymmetric unit	2	2	4	2	1
Total number of observations*	567,964 (28,298)	230,162 (15,688)	161,907 (24,828)	559,086 (26,040)	207,502 (9011)
Number of unique reflections*	42,948 (2306)	30,917 (2138)	23,519 (3629)	76,833 (3573)	29,292 (1327)
Completeness (%)*	99.5 (91.7)	99.5 (93.3)	99.1 (94.5)	99.6 (94.5)	97.1 (89.0)
Redundancy*	13.2 (12.3)	7.4 (7.3)	6.9 (6.8)	7.3 (7.3)	7.1 (6.8)
I/σ(I)*	19.1 (1.4)	16.1 (1.7)	7.2 (1.1)	13.2 (1.2)	10.5 (3.0)
Rmerge*	0.055 (1.357)	0.050 (0.857)	0.163 (1.182)	0.077 (1.102)	0.110 (0.822)
Rmeas*	0.057 (1.416)	0.053 (0.921)	0.192 (1.393)	0.083 (1.187)	0.120 (0.897)
Rpim*	0.016 (0.395)	0.019 (0.330)	0.102 (0.732)	0.031 (0.433)	0.046 (0.353)
CC(1/2)*	1.000 (0.693)	1.000 (0.779)	0.996 (0.710)	0.999 (0.633)	0.994 (0.561)
**Refinement**					
Number of reflections used in refinement*	42,845 (2557)	30,859 (2634)	23,457 (2711)	76,701 (2494)	29,234 (2731)
Resolution (Å)*	43.98–1.79 (1.83–1.79)	43.98–2.00 (2.07–2.0)	44.95–2.88 (3.01–2.88)	43.82–1.46 (1.49–1.47)	29.64–1.47 (1.52–1.47)
R_work_/R_free_	0.196/0.224 (0.30/0.372)	0.203/0.242 (0.32/0.330)	0.208/0.270 (0.331/0.365)	0.188/0.209 (0.303/0.336)	0.186/0.212 (0.228/0.275)
CC(work)*	0.924 (0.813)	0.93 (0.805)	0.95 (0.638)	0.933 (0.706)	0.937 (0.849)
No. of atoms (excluding H)	3181	3093	5956	3623	1778
Protein	2952	2956	5908	3139	1565
Solvent	144	101	38	436	204
Ligands	85	36	10	48	9
Protein Residues	374	374	751	397	200
**RMSD from ideal geometry**					
Bonds (Å)	0.007	0.008	0.003	0.005	0.006
Angles (°)	1.16	0.95	0.47	0.8	0.78
**Ramachandran (%)**					
Favoured	97.57	97.84	96.37	98.97	98.98
Allowed	2.43	2.16	3.63	1.03	1.02
Outliers	0	0	0	0	0
Rotamer Outliers (%)	1.26	1.26	1.11	1.48	0.6
Clashscore (%)	3.49	2.63	2.5	7.81	5.69
Average B-factors	39.6	47.3	76.9	25.4	18.4
Protein	39.3	47.28	76.88	23.96	17.18
Ligands	45.1	44.85	86.77	40.93	30.94
Solvent	42.53	47.54	55.67	33.81	26.81

^*^Values in parentheses represent the highest resolution shell.

Local adjustments in Loop 1 (F63–M64–G65–G66), which links the DsbA TRX domain to the inserted helical domain^19^ (Fig. 2C), allowed accommodation of alternative orientations. Structural overlays with apo DsbA (PDB: 1FVK) showed that the overall fold was unchanged, but F63 and M64 shifted outward by up to 12 Å, opening the active-site cavity to accommodate side chains such as the Cys+2 leucine of LptD (Fig. 2C, Supplementary Movie 1). This local flexibility, absent in the apo structure, supports a role for Loop 1 in substrate adaptation. Hydrophobic contacts varied between binding modes (Supplementary Fig. 1), suggesting that peripheral plasticity allows substrate diversity.

### A conserved cis-proline loop anchors the substrate cysteine

Despite the observed differences in peptide orientations, both complexes shared a common anchoring mechanism mediated by the *cis-*proline loop (R148–G149–V150–*cis*P151) (Fig. 2D*,* E). Two backbone hydrogen bonds from V150 engaged the substrate cysteine’s amide and carbonyl (3.0 and 2.8 Å, respectively), while the carbonyl of R148 hydrogen-bonded to the amide of the Cys+2 substrate residue. Additional stabilisations arose from the P151 carbonyl interacting with the side chain of the Cys-1 substrate residue, and in binding mode II, a fifth hydrogen bond was observed between the R148 carbonyl and the amide of the Cys+3 substrate residue (3.2 Å) (Fig. 2D*,* E).

This invariant hydrogen-bond network imposed a strict C-to-N orientation of the substrate peptide, opposite to the N-to-C direction of the DsbA backbone. In this arrangement, the enzyme and substrate cysteines align to form a right-handed disulfide (χ₃ ≈ +90°, Fig. 2F, G), a conformation that minimises torsional strain and orients the S–S antibonding orbital (σ*) approximately perpendicular to the bond axis. As such, this geometry positions the σ* of the leaving sulphur antiperiplanar (≈180°) to the lone pair of the incoming resolving cysteine, providing the optimal stereo electronic overlap for SN2-like thiol–disulfide exchange. The *cis*-proline loop, therefore, not only stabilises the mixed-disulfide intermediate but also locks it into a geometry that facilitates its rapid resolution.

### Structural comparison of DsbA–substrate complexes highlights a conserved catalytic scaffold

**Fig. 3:**
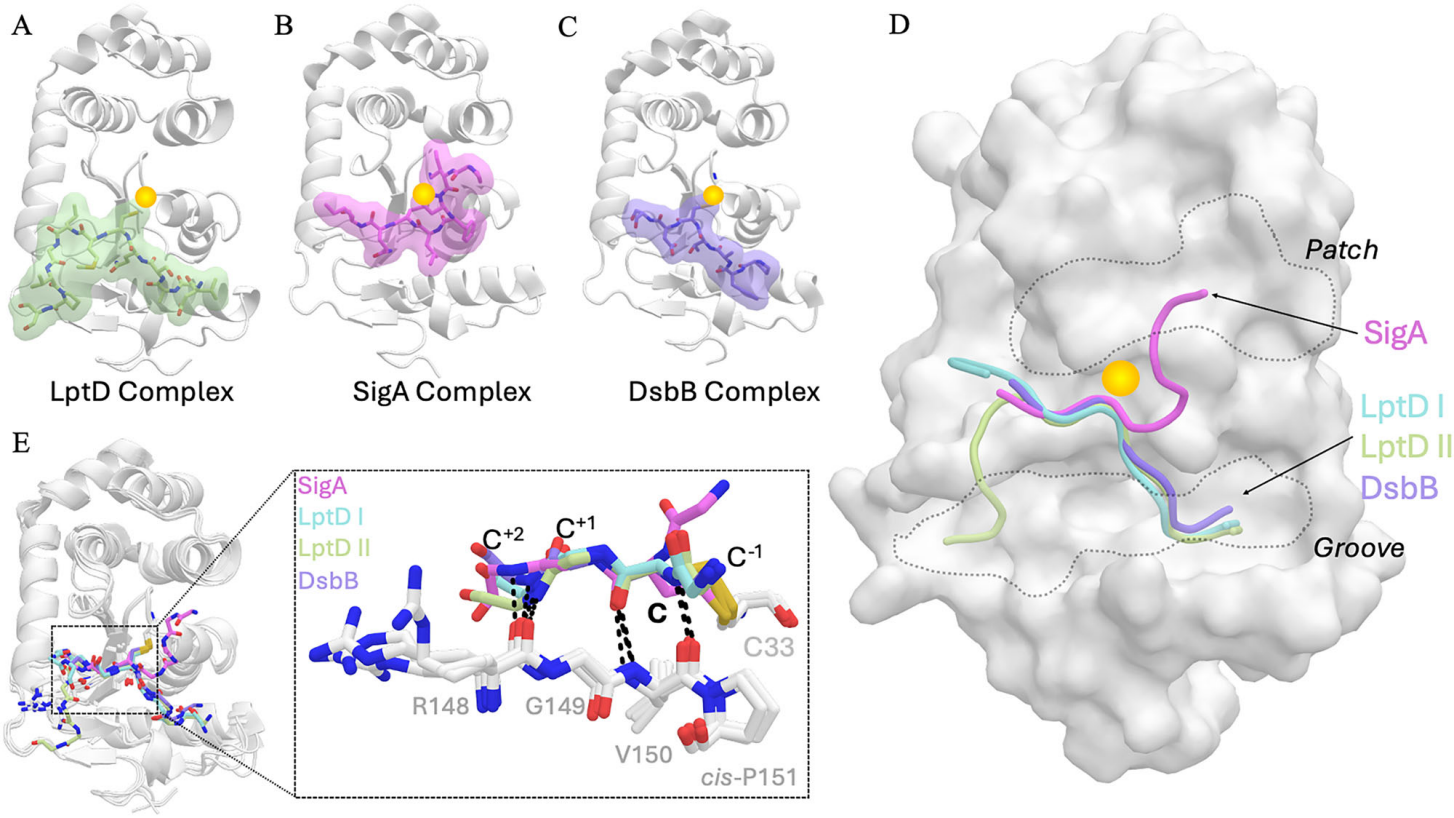
Comparison of DsbA complex structures. **A**–**C** Structures of DsbA bound to different peptides, shown in white with catalytic cysteines in yellow. The SigA peptide (PDB: 3DKS)^40^ (pink) interacts with both TRX and helical domains (**A**), while the LptD peptide (green) penetrates the TRXdomain hydrophobic groove (**B**), similar to the DsbB peptide (purple; PDB: 4TKY)^43^ (**C**). **D** Overlay of bound peptides highlights alternative orientations: SigA (pink) lies across the active-site surface, whereas LptD (blue/green) and DsbB (purple) project into the groove. **E** Conserved hydrogen-bonding network mediated by the *cis*-proline loop. Across all complexes, the substrate cysteine (labelled C) is fixed by two hydrogen bonds from V150 (its carbonyl oxygen to the substrate cysteine amide, and its amide nitrogen to the substrate cysteine carbonyl) and one from R148 (its carbonyl oxygen to the backbone amide of the substrate C+2 residue), establishing an invariant scaffold for catalysis despite variation in peripheral contacts.

We next compared the DsbA-LptD complexes with the only previously reported substrate-bound DsbA structure, the DsbA-SigA peptide complex (PDB: 3DKS), which represents, to our knowledge, the sole available example of DsbA captured with a physiologically relevant substrate^40^. In that structure, the SigA peptide, tethered via a thioether bond, adopts a conformation that primarily engages the hydrophobic patch above the catalytic C–P–H–C motif formed by DsbA helical domain, without entering the hydrophobic groove (Fig. 3A). By contrast, the LptD peptide binds within the TRX domain, deeply engaging the hydrophobic groove (Fig. 3B), a mode resembling DsbA-DsbB complexes (PDBs: 2ZUP, 2HI7^41,42^), which is the only structure of DsbA bound to a full-length protein, as well as a DsbB-derived synthetic peptide (PDB: 4TKY^43^) (Fig. 3C). These comparisons suggest that the helical domain contributes only to certain substrate interactions, while the TRX core provides the common catalytic platform shared across the superfamily.

Despite differences in peripheral contacts, all DsbA complexes converge on a conserved active-site architecture (Fig. 3D*,* E). In each case, the substrate cysteine is anchored by two backbone hydrogen bonds from V150, positioned immediately before *cis*P151, and reinforced by a third bond from the R148 carbonyl to the substrate Cys+2 residue. These recurring interactions provide a precise hydrogen-bonding scaffold that fixes the cysteine in-line for thiol–disulfide exchange (Fig. 3E).

While peptide-based substrates cannot be assumed to fully recapitulate the complexity of interactions formed by intact protein substrates, they nevertheless provide valuable mechanistic insight. Indeed, DsbA engages substrates during translocation to the periplasm when proteins are unfolded or partially folded, and recognition is dominated by exposed cysteine-containing segments rather than fully folded domains. In this context, peptide substrates provide a relevant approximation of the reactive state encountered during catalysis, capturing key features of mixed-disulfide formation and cysteine positioning, even if additional contacts contribute to substrate recognition in full-length proteins.

Together, these features establish the *cis*-proline loop as a structural lock that enforces catalytic geometry and dictates substrate orientation, even as peripheral binding varies. This arrangement allows DsbA to accommodate chemically diverse substrates through flexible contacts while maintaining catalytic precision. The *cis* configuration of P151, supported by the local flexibility of G149, is central to this mechanism.

### Conservation of cis-proline loop architecture across DsbA enzymes

**Fig. 4:**
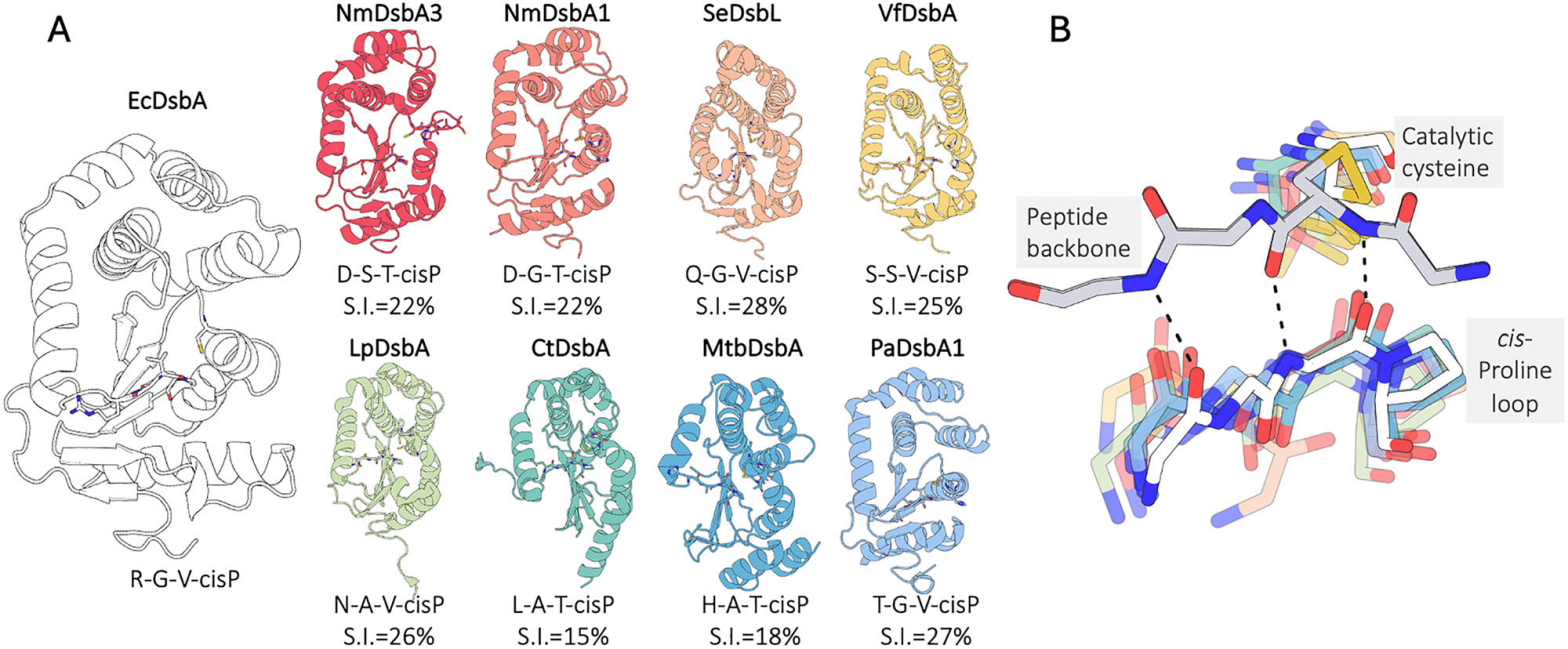
Conservation of *cis*-proline loop architecture across bacterial DsbAs. **A** Cartoon representations of diverse DsbA homologues, from *E. coli* (EcDsbA, PDB: 1FVK^26^), *Neisseria meningitidis* (NmDsbA3, PDB: 3DVX^62^; NmDsbA1, PDB: 3DVW^62^), *Salmonella enterica* (SeDsbL, PDB: 3L9U^63^), *Vibrio fischeri* (VfDsbA, PDB: 3FEU), *Legionella pneumophila* (LpDsbA, PDB: 4JRR), *Chlamydia trachomatis* (CtDsbA, PDB: 5KBC^64^), *Mycobacterium tuberculosis* (PDB: 4K6X^65^), and *Pseudomonas aeruginosa* (PaDsbA1, PDB: 3H93^66^). Active-site CXXC motifs and *cis-*proline loops are shown as sticks. The *cis*-proline loop sequences and percent identity to *E. coli* DsbA are indicated. **B** Overlay of *cis-*proline loops from all homologues reveals identical backbone geometry relative to the catalytic cysteine, despite variation in loop sequence.

To determine whether the *cis*-proline loop geometry seen in *E. coli* DsbA complexes is conserved, we compared high-resolution structures from diverse bacterial DsbA homologues (Fig. 4A). Despite substantial sequence variation, all structures exhibited the same backbone geometry of the *cis*-proline loop relative to the catalytic cysteine (Fig. 4B).

The *cis*-proline residue was strictly conserved, and the position equivalent to *E. coli* DsbA G149 was always occupied by a small residue (Gly, Ala, Ser, or Thr), likely preserving local flexibility and avoiding steric clashes near the active site. Similarly, the position equivalent to V150 was usually Val or Thr, maintaining a compact side chain in the scaffold. The R148-equivalent residue showed more sequence diversity but retained the backbone conformation required to form the invariant hydrogen-bond network.

Together, these trends define a concise *cis*-proline loop consensus characterised by a variable residue at position −3, a small residue at position −2, a compact residue at position −1, and an invariant *cis*-proline that together preserve the backbone geometry required for substrate cysteine anchoring (consensus motif: X–[G/A/S/T]–[V/T]–P(*cis*)).

Collectively, these comparisons show that sequence identities vary widely across DsbAs, but the rigid backbone scaffold of the *cis-*proline loop is invariant. This conserved geometry underpins the ability of DsbA enzymes to maintain catalytic precision while evolving diverse substrate-binding surfaces.

### Functional consequences of cis-proline loop mutations

To probe the role of the conserved *cis-*proline loop, we mutated G149 and *cis*P151 in DsbA, residues predicted to maintain loop geometry and the hydrogen-bonding platform. Specifically, G149 was replaced with Met, Val, Thr, or Lys, while *cis*P151 was substituted with Thr, a mutation previously linked to substrate trapping^36,44^.

**Fig. 5:**
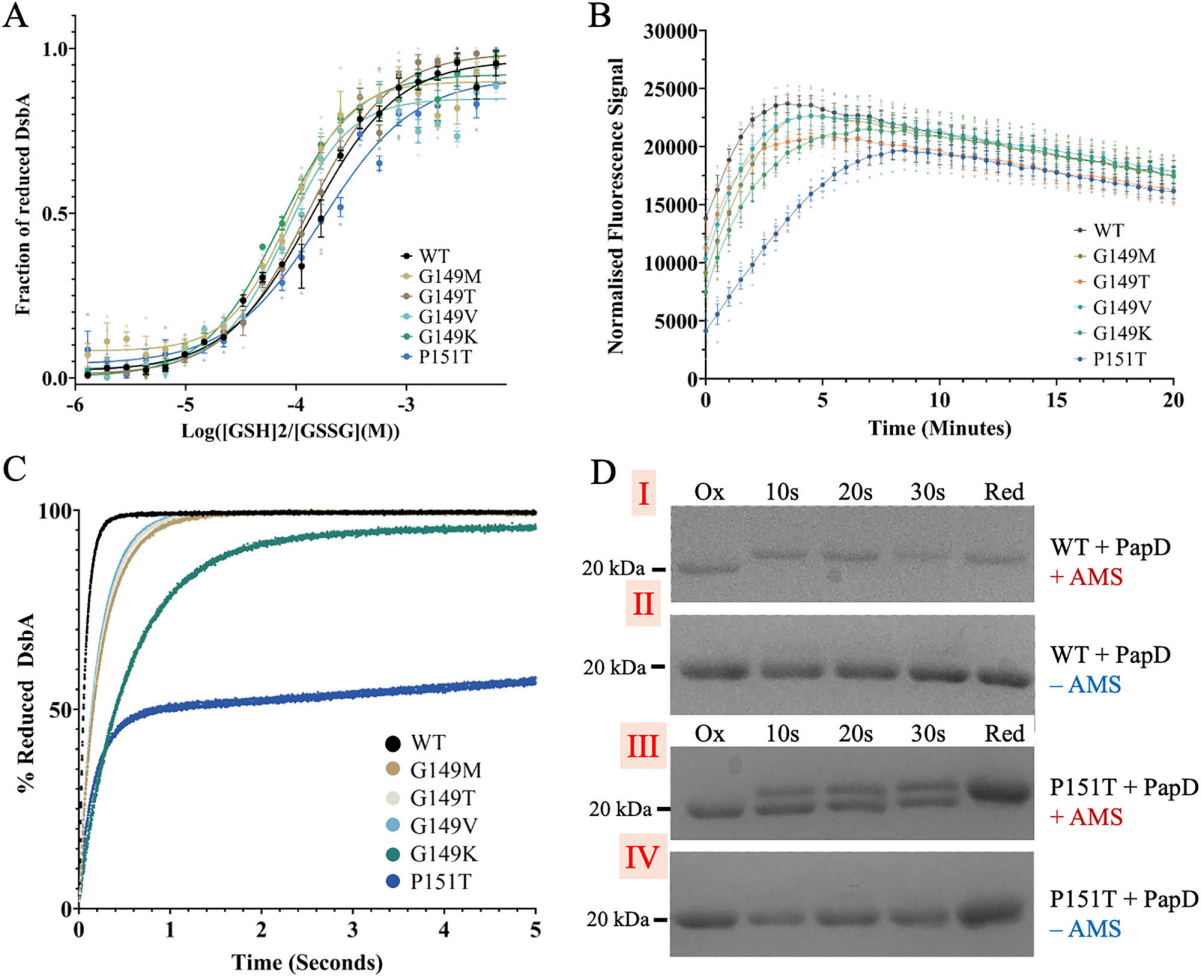
Functional consequences of *cis*-proline loop mutations in DsbA. **A** Redox potentials of wild-type and loop variants. G149 substitutions shifted potentials to more oxidising values, while P151T resembled the wild-type. Measurements were performed in triplicate on three independent occasions; a representative dataset shown. **B** Peptide oxidation assays showed near-wild-type turnover for G149M/V, reduced activity for G149T/K, and severe impairment for P151T. Data are mean ± SEM from *n* = 3 biologically independent experiments, each measured in technical triplicate. **C** Stopped-flow kinetics confirmed partial activity for G149 mutants and incomplete substrate oxidation by P151T. Measurements were performed in triplicate on three independent occasions; representative dataset shown. **D** Biochemical analysis of wild-type and P151T DsbA during reaction with the PapD peptide. (I–II): Samples of wild-type DsbA were analysed in parallel with AMS to assess redox state or without AMS to detect potential trapped DsbA–PapD intermediates. Wild-type DsbA efficiently transitioned to the reduced state following substrate incubation, with no trapped intermediates detected. (III–IV): show the corresponding reactions with the P151T mutant. AMS labelling revealed that a substantial fraction of P151T (~40%) remained oxidised after incubation with substrate, indicating a failure to efficiently bind and oxidise the substrate. No stable mixed-disulfide intermediates were observed in the absence of AMS.

Redox titrations^45^ revealed that G149 substitutions increased the redox potential relative to wild type (–123 mV), with G149M, G149V, and G149K shifting the potential to less negative values, whereas G149T was essentially unchanged (Fig. 5A, Supplementary Fig. 2, Table 2). By contrast, P151T retained a wild-type-like redox potential, in contrast to earlier reports^44^, likely reflecting methodological differences.

**Table 2:** Redox and kinetic properties of DsbA mutants

Variant	*K*_*eq*_ (M)	Redox Potential (mV)	Rate Constant (M^−1^ s^−1^) (×10^5^)	Relative activity (%)
Wild type	1.33 ± 0.00615 × 10^−4^	−123 ± 0.06	4.08 ± 0.008	100%
G149M	7.99 ± 0.379 × 10^−5^	−117 ± 0.6	2.05 ± 0.021	50.25%
G149T	1.60 ± 0.0854 × 10^−4^	−122 ± 1	2.14 ± 0.024	52.45%
G149V	8.22 ± 0.210 × 10^−5^	−103 ± 0.3	2.08 ± 0.031	50.98%
G149K	6.61 ± 0.515 × 10^−5^	−115 ± 0.3	0.91 ± 0.063	22.33%
P151T	1.76 ± 0.146 × 10^−4^	−126 ± 1	1.48 ± 0.039	36.27%

Redox potentials are reported as mean ± SEM. Second-order rate constants are reported as mean ± SEM, with activities expressed relative to wild-type DsbA.

Values are mean ± SEM from *n* = 3 biologically independent experiments.

Despite modest changes in redox potential, all variants showed catalytic defects. In FRET-based peptide oxidation assays, G149M and G149V supported near-wild-type turnover, while G149T and G149K oxidised substrates more slowly (Fig. 5B). In contrast, P151T exhibited severely impaired turnover and failed to fully oxidise the peptide substrate.

Stopped-flow kinetics provided a quantitative framework to interpret these trends. Although G149V, G149T, and G149M all supported near-wild-type turnover in endpoint peptide oxidation assays, kinetic analysis revealed that these variants oxidised a PapD-derived peptide^24^ with similar ~2-fold reduced rate constants relative to wild-type DsbA (4.1 × 10^5^ M^−1^ s^−1^), clustering within a narrow range (22–52% of wild type) (Fig. 5C, Table 2, Supplementary Fig. 3*A*).

Notably, G149M, G149V, and G149T exhibit comparable kinetic penalties despite markedly different side-chain sizes and chemistries, indicating that side-chain size per se is not the primary determinant of activity at this position. Instead, substitution at G149 modulates catalytic efficiency through chemical and microenvironmental effects, including polarity, hydrophobicity, and charge, that influence substrate binding, mixed-disulfide formation, and resolution. The more pronounced defect observed for G149K is consistent with an additional kinetic penalty imposed by the introduction of a charged side chain.

The P151T variant displayed a distinct kinetic phenotype. Although its redox potential was comparable to that of wild-type DsbA, P151T exhibited a markedly reduced apparent rate constant (approximately 36% of wild type) and plateaued at ~50% turnover, indicating inefficient completion of the catalytic cycle (Fig. 5C). To determine whether this defect reflected altered substrate handling, we examined the redox behaviour of wild-type and P151T DsbA during reaction with the native PapD peptide substrate. Wild-type DsbA efficiently transitioned to the reduced state following substrate incubation, as assessed by AMS labelling, with no evidence of trapped mixed-disulfide intermediates (Fig. 5D, panels I–II, Supplementary Fig. 3B). In contrast, although no stable mixed-disulfide species were detected for the P151T variant, a substantial fraction of the protein (~40%) remained oxidised after incubation with substrate (Fig. 5D, panels III–IV). These observations indicate that the P151T substitution compromises productive substrate engagement and/or efficient disulfide transfer, providing a biochemical explanation for the incomplete turnover observed in kinetic assays.

Together, these results show that G149 functions as a tuning position that modulates catalytic efficiency through chemical effects independent of redox thermodynamics, while *cis*P151 is essential to maintain the *cis* geometry that enforces catalytic efficiency and full substrate oxidation. The *cis*-proline loop, therefore, combines chemical tuning with structural rigidity, explaining its strong conservation across thioredoxin-fold enzymes.

### Structural basis of impaired catalysis in loop variants

To understand the biochemical defects caused by loop mutations, we solved crystal structures of three representative variants: P151T (impaired catalysis), G149K (highest redox potential and lowest activity), and G149T (minimal redox change but reduced efficiency). The structures were refined to 2.88, 1.79, and 2.00 Å resolution, respectively (Table 1).

**Fig. 6:**
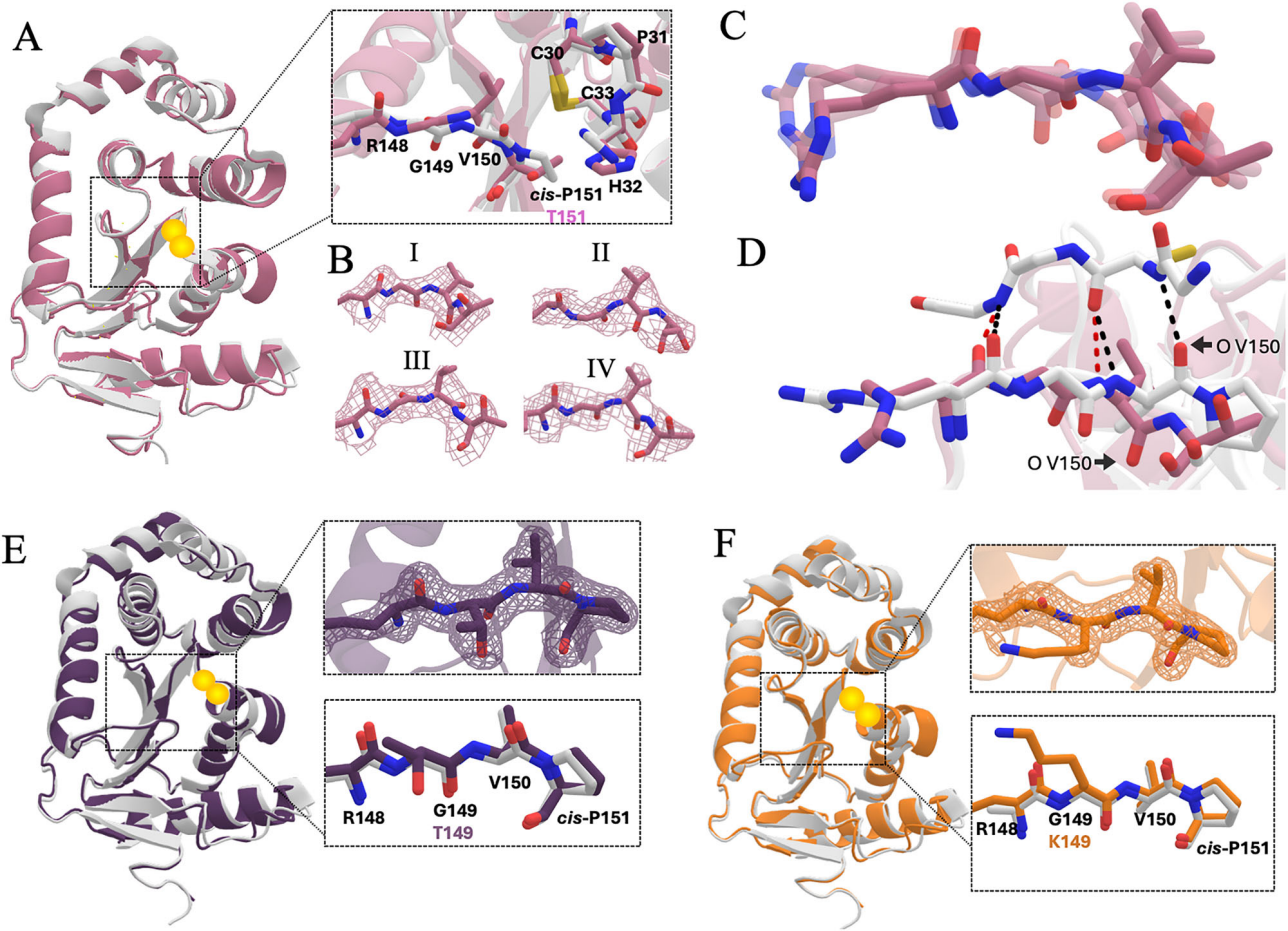
Structural basis of impaired catalysis in *cis*-proline loop variants. **A** Overlay of wild-type DsbA (white) with P151T (pink). Catalytic cysteines are shown as yellow spheres. Inset: changes at the active site induced by the P151T substitution. **B** Loop disruption with *cis*-to-*trans* isomerisation. Each protomer in the asymmetric unit is shown in pink, with the 2mF_o_-DF_c_ map contoured at 1.0 σ. **C** Overlay of active sites from the four P151T protomers, highlighting backbone shifts and multiple conformations of the V150 carbonyl. **D** Displacement of V150 carbonyl in P151T (pink) abolishes hydrogen-bonding anchors and clashes with modelled substrate (white). Overlays of native DsbA (white) with G149T (purple, **E**) and G149K (orange, **F**). Insets show the *cis-proline* loop of each mutant with electron density (2mFo-DFc maps contoured at 1.0σ) and the corresponding loop overlays with the native structure.

For P151T, the overall fold closely matched wild-type DsbA^26^ (RMSD ~ 0.36 Å over 155 Cα atoms), but the *cis*-proline loop was markedly perturbed (Fig. 6A). The *cis*-to-*trans* isomerisation abolished the canonical geometry: the V150 carbonyl was displaced from ~3.8 Å to 5.6–6.4 Å from Cys30, eliminating the hydrogen-bonding platform required for substrate cysteine anchoring (Fig. 6B–D). Elevated B-factors indicated increased flexibility (Supplementary Movie 2), and in some protomers the V150 carbonyl projected into the substrate-binding cleft, introducing steric clashes that would compromise peptide accommodation (Fig. 6D). These features explain why, although the P151T mutant retains some catalytic activity, it fails to efficiently complete substrate oxidation.

In contrast, G149T and G149K closely resembled wild-type (RMSD 0.48–0.50 Å over 172 Cα atoms) and preserved the canonical *cis-*proline loop geometry (Fig. 6E, F). For G149T, the absence of major structural rearrangements is consistent with its near-native redox potential. In G149K, introduction of a positively charged side chain adjacent to Cys30 likely stabilises the catalytic thiolate, accounting for the observed increase in redox potential (Supplementary Fig. 4).

Despite preservation of oxidising redox potential and loop geometry in both variants, G149T and G149K display reduced catalytic efficiency toward the peptide substrates used in this study. Replacement of Gly149, which lacks a side chain, with either a polar side chain (G149T) or a charged side chain (G149K) introduces additional steric constraints and alters the local chemical environment, potentially affecting short-range steric, hydrogen-bonding, and electrostatic interactions with the peptide substrate. These changes are expected to restrict local conformational sampling and may impair productive substrate engagement and turnover, without disrupting the underlying *cis*-proline loop architecture.

Together, these structures rationalise the biochemical findings: substitutions at G149 preserve *cis*-proline loop geometry and redox competence but reduce catalytic efficiency through perturbation of the local active-site environment. In contrast, substitution of P151 disrupts the *cis*-loop geometry itself, abolishing the hydrogen-bonding platform required for precise cysteine positioning and efficient completion of the catalytic cycle.

## Discussion

Thioredoxin-fold oxidoreductases achieve remarkable catalytic speed while acting on chemically diverse substrates, yet the basis of this long-standing precision-versus-diversity paradox has remained unresolved. Here, we show that a conserved *cis*-proline lock adjacent to the CXXC motif provides a unifying catalytic feature.

Structural snapshots of transient DsbA–substrate complexes reveal that backbone hydrogen bonds from this loop clamp substrate cysteine into a right-handed disulfide, an optimal geometry for thiol–disulfide exchange, while surrounding flexibility enables recognition of diverse substrates.

Mutational analysis confirmed the pivotal role of this loop. Residue *cis*P151 is essential for maintaining the *cis* geometry, and its substitution disrupts the scaffold, reduces the kinetic rate, and prevents complete substrate turnover. In contrast, changes at Gly149 preserve the geometry but alter the local environment, incurring a measurable kinetic penalty in peptide oxidation. This penalty cannot be attributed to altered thermodynamics or disruption of the catalytic scaffold and instead reflects perturbation of the local active-site microenvironment. Accordingly, variation at this position across DsbA homologues is likely to function as a tuning element that modulates substrate specificity rather than general catalytic competence.

Comparative structural analysis reveals that this architecture is conserved across diverse DsbA homologues, despite wide sequence variation. Moreover, surveys of thioredoxins, protein disulfide isomerases, peroxiredoxins, and related enzymes using FoldDisco^46^, identified complexes across bacteria, yeast, mammals, and parasites, with the same invariant scaffold adjacent to the CXXC motif (Supplementary Table 1, Supplementary Fig. 5). The recurrence of this loop and its conserved geometry across the superfamily highlights strong evolutionary pressure to maintain catalytic fidelity^47^, even at the folding cost of a *cis-*proline^48,49^.

Beyond this rigid core, TRX-fold enzymes diversify substrate recognition through auxiliary domains and adaptable surface features. In DsbA, for example, the TRX domain provides the catalytic lock, while the inserted helical domain and flexible loops allow binding of chemically varied partners. Similar modularity is evident across the wider superfamily, and these additional interactions that stabilise enzyme–substrate intermediates likely explain why TRX-fold enzymes turnover protein substrates more efficiently than thiol/disulfide–containing compounds^16,50^.

**Fig. 7:**
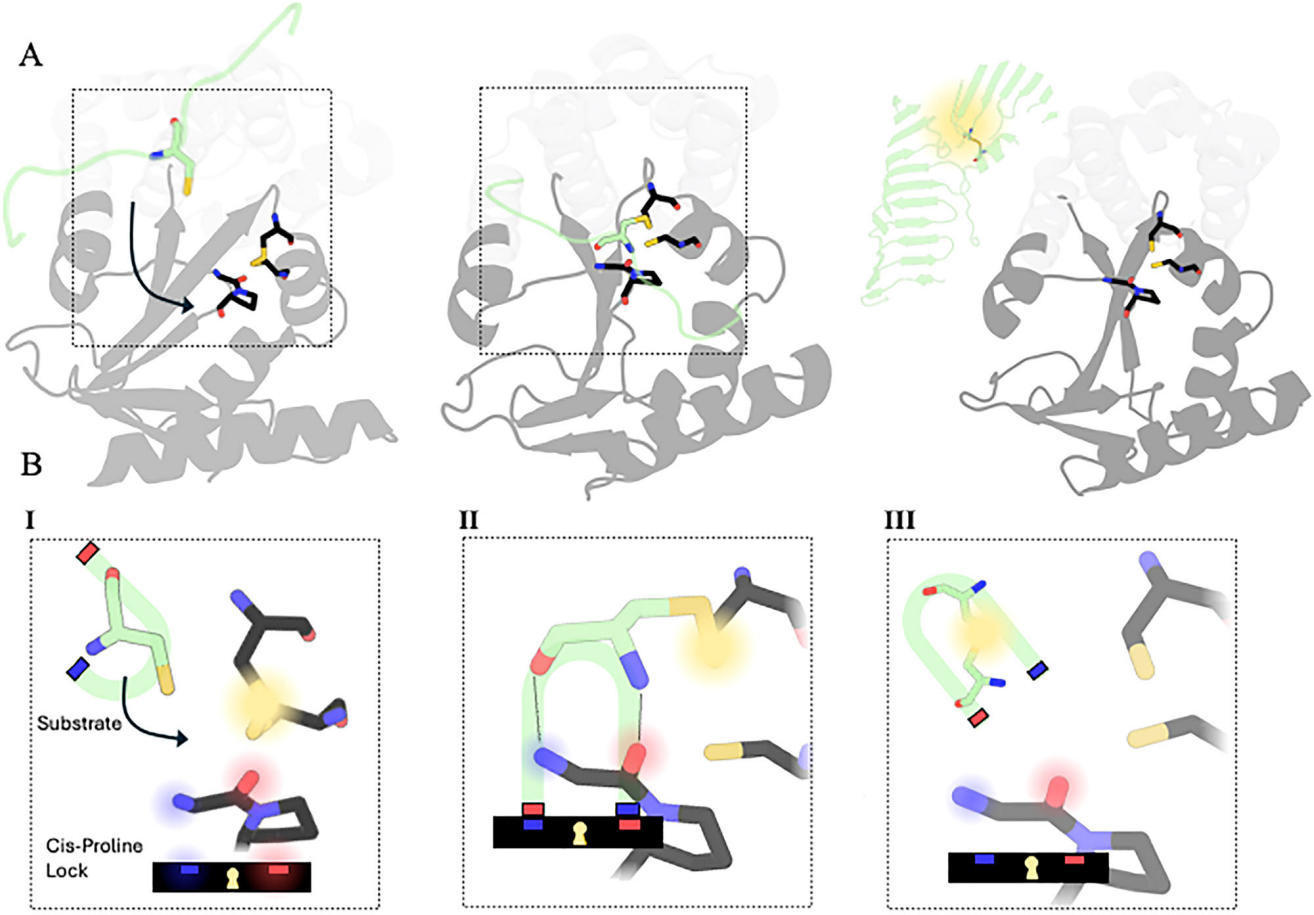
Mechanistic model of the *cis*-proline lock. **A** Cartoon representations illustrate *E. coli* DsbA with the TRX domain (light grey), catalytic cysteines and *cis*-proline loop (dark grey), and substrate peptide (green). **B** Schematic diagrams depict the molecular lock logic: (I) loop positioning of the substrate cysteine, (II) clamping of the mixed disulfide, and (III) unlocking by resolution through a second cysteine. The *cis*-proline loop acts as a molecular lock that enforces catalytic geometry in TRX-fold enzymes. Backbone hydrogen bonds from the loop clamp the substrate cysteine into a right-handed disulfide, the productive geometry for thiol–disulfide exchange. Resolution of the mixed disulfide releases the product: in TRX oxidases, this is achieved by a second cysteine from the substrate, whereas in TRX reductases, it is provided by the second cysteine of the enzyme’s CXXC motif. This lock-and-key mechanism explains how thioredoxin-fold enzymes maintain catalytic precision, while protein modularity enables broad substrate diversity.

In summary, although TRX-fold proteins have diversified to expand their functional range in different cellular or ecological contexts—one invariant feature is the *cis*-proline lock, which underpins catalytic fidelity across the entire superfamily (Fig. 7). This conserved anchoring mechanism enforces a productive catalytic geometry while chemically tuneable elements within and around the loop modulate substrate engagement and turnover, conferring broad substrate diversity. Such a dual strategy explains both the evolutionary success of the TRX-fold and its adaptation to diverse redox processes. These insights establish a framework for engineering oxidoreductases with tailored activity and for developing inhibitors targeting thioredoxin-like proteins in infection, cancer, and neurodegeneration.

## Methods

### Mutagenesis, expression and purification

*E. coli dsbA*, without its signal sequence, was cloned into pMCSG11^51^. Primers for site-directed mutagenesis (G149V, G149T, G149M & G149K, P151T and C33A) were obtained from Sigma Aldrich (Supplementary Table 2). Mutants were generated using the Q5® Site-Directed Mutagenesis Kit (NEB) and subsequently sequenced (Macrogen). Proteins were expressed in BL21(DE3) cells using an autoinduction method for 24 h at 30 °C in media supplemented with ampicillin (100 μg/mL)^52^. Resulting cells were harvested and resuspended in Tris buffer (50 mM Tris, pH 7.5, 150 mM NaCl). Following resuspension, cells were sonicated to induce lysis (Misonix S-4000 Ultrasonic Liquid Processor (Qsonica)). N-terminal 6xHis-tagged constructs were purified using nickel affinity chromatography followed by TEV-cleavage and reverse nickel affinity chromatography. Resulting samples were oxidised using a 20-fold molar excess of oxidised glutathione (GSSG) (1 h at 4 °C) prior to final purification *via* size exclusion chromatography using a Superdex 75 HiLoad 16/600 column (GE HealthCare) equilibrated in 50 mM HEPES, pH 8.5, 100 mM NaCl. Eluted fractions were analysed by sodium dodecyl sulphate-polyacrylamide gel electrophoresis (SDS–PAGE) to assess purity.

### Redox potential assay

The redox potential of *E. coli* DsbA variants was determined as follows^45^: proteins (3 µM) were equilibrated at 25 °C in 10 mM sodium phosphate, pH 7.0, 0.1 mM EDTA with 1 mM oxidised glutathione (GSSG) and varying concentrations of reduced glutathione (GSH) (0.036–1.22 mM) for 24 h. The fraction of reduced protein was measured by intrinsic tryptophan fluorescence (excitation 280 nm, emission 332 nm) in a Spectramax M5e plate reader (Bio-strategy) using a 96-well half-area black plate (Greiner). Equilibrium data were fitted^53^ in GraphPad Prism v10 to:

*Z* = ([GSH]^2^/[GSSG]) / (K_eq_ + ([GSH]^2^/[GSSG]))

Where *Z* is the fraction of reduced protein at equilibrium. The redox potential was then derived using the Nernst equation,

*E*^0^′_DsbA_ = *E*^0^′_GSH/GSSG_ – (RT/2*F*) ln K_eq_

Where *E*^0^’_GSH/GSSG_ is the standard potential of −240 mV, R is the universal gas constant 8.314 J K^−1^ mol^−1^, T is the absolute temperature in K, F is the Faraday constant 9.648 × 104 C mol^−1^, and K_eq_ is the equilibrium constant. Values reported are the mean ± SEM from three independent experiments (Supplementary Fig. 2).

### Peptide oxidation assay

A fluorescence resonance energy transfer (FRET)-based assay was utilised to determine the activity of the DsbA mutants against target substrates in vitro^33,54^. A peptide derived from *E. coli* DsbL substrate ASST (CNENGLCK)^55^ labelled with an N-terminal europium DOTA (1,4,7,10-tetraazacyclododecane−1,4,7,10-tetraacetic acid) and C-terminal coumarin amide group was utilised as the target substrate. Peptide oxidation by DsbA results in cyclisation of the peptide that brings the Eu-DOTA and methyl coumarin into proximity, triggering fluorescence excitation of the coumarin moiety at 340 nm and fluorescence emission at 615 nm. Reactions were performed in 50 µL volumes containing 500 nM protein, 2 mM GSSG, 15 µM ASST, diluted from a 2 mM ASST stock prepared in 100 mM imidazole, pH 6.0. Assays were performed using a PerkinElmer 384-well white opaque microplate and measured using time-resolved fluorescence at an excitation λ = 340 nm and emission λ = 615 nm, and measured over 20 min on a CLARIOstar plate reader (BMG Labtech). Assays were performed in triplicate and analysed using GraphPad Prism v10.

### Stopped-flow kinetics assay

The rapid kinetics of DsbA-catalysed oxidation of a PapD-derived peptide (Ac-FICNFSRCSV-NH₂) were monitored using an Applied Photophysics SX20 stopped-flow spectrometer^24^. Intrinsic tryptophan fluorescence of DsbA decreases upon reduction of the active-site disulfide^33^, allowing direct monitoring of the redox state of the enzyme during catalysis. Before measurements, DsbA variants were diluted to 2 µM in 20 mM HEPES, pH 7.5, 100 mM NaCl, and 0.1 mM EDTA. To determine the fully reduced fluorescence baseline, each variant was mixed with 2 µM dithiothreitol (DTT) at 25 °C, and the change in emission at 330 nm (λ_ex_ = 295 nm) was recorded over 40 s using a λ_em_ 330 nm emission filter.

For kinetic measurements under second-order conditions, oxidised DsbA (2 µM) was rapidly mixed with PapD peptide (4 µM) at 25 °C, and the decrease in fluorescence was recorded over time. This decrease corresponds to the reduction of DsbA during formation of the transient mixed-disulfide intermediate and its subsequent resolution. Initial rates were calculated from the linear portion of the fluorescence reaction curve using Pro-Data SX software (Applied Photophysics). All reactions were performed in triplicate, and rate constants are reported as mean ± SEM from three independent experiments.

### P151T mixed-disulfide trapping assay

The DsbA P151T variant was tested to assess its ability to form a mixed-disulfide adduct with a PapD peptide (Ac-FICNFSRCSV-NH₂). Oxidised DsbA P151T (20 µM) was prepared in 20 mM HEPES pH 7.0, 100 mM NaCl, 0.1 mM EDTA, and rapidly mixed with reduced PapD peptide at a 1:10 (protein:peptide) molar ratio in a final volume of 25 µL at 25 °C. Reactions were quenched with trichloroacetic acid (TCA) at 10, 20, and 30 s.

Quenched samples were processed using two parallel workflows. For adduct formation detection, TCA pellets were washed with cold acetone, resuspended in non-reducing Laemmli buffer, and analysed by SDS–PAGE. The resulting SDS–PAGE gel for the trapped DsbA P151T–PapD mixed-disulfide adduct is detected due to a slower migrating band approximately 1.2 kDa above the P151T sample, consistent with the mass of the ~10-residue peptide. For assessment of the redox state across the reaction, TCA pellets were resuspended in AMS buffer (2 mM AMS, 50 mM Tris, pH 7.0, 2% SDS) and incubated at room temperature for 30 min prior to the addition of non-reducing sample buffer and SDS–PAGE. The resulting samples on SDS–PAGE would lead to a mobility shift of ~0.5 kDa per thiol, leading to the reduced P151T (two thiols) migrating ~1 kDa slower than oxidised DsbA, while a disulfide-linked P151T–PapD adduct, lacking free cysteines at the active site, does not exhibit an AMS shift.

### *E. coli* DsbA - peptide complex formation

For complex formation, a C33A mutant of DsbA was used to stabilise the reaction. The purified DsbA_C33A was fully reduced with DTT overnight at 4 °C. A PD MiniTrap desalting column equilibrated with 25 mM HEPES, pH 6.8, 50 mM NaCl was used to remove excess reducing agent. Reduced DsbA_C33A was diluted to 0.1 mM and mixed with 5,5′-dithio-bis-(2-nitrobenzoic acid) (DTNB) powder (5 mM) and incubated for 1 h at 25 °C. Following incubation, the protein was further equilibrated using a PD MiniTrap desalting column to isolate DTNB-conjugated DsbA_C33A samples. Lyophilised peptides were then mixed at a 5:1 ratio of (peptide: protein) and incubated at 4 °C for 15 h.

Successfully conjugated DsbA_C33A-peptide samples were isolated using hydrophobic interaction chromatography (HIC). Prior to HIC, 0.8 M ammonium sulphate was added to the incubated complex sample and incubated for 1 h with gentle stirring. The sample was then eluted on a gradient of 1–0 M Ammonium sulphate. The purified complex was buffer exchanged into 25 mM HEPES, pH 7.

### Crystallisation and data collection

Crystallisation conditions for mutant and complex structures were first identified by high-throughput screening using the sitting-drop vapour diffusion method (0.2 µL drops) with a Crystal Gryphon Crystallisation Robot (Art Robbins Instruments, Sunnyvale, CA, USA) at 293 K. Optimised conditions for each construct are listed in Supplementary Table 3. The DsbA P151T mutant was additionally crystallised in 2 µL (1:1 protein to reservoir) drops in 24-well plates with 500 µL reservoirs. All crystals were cryoprotected in 20% (v/v) glycerol diluted in mother liquor and flash-cooled in liquid nitrogen before data collection.

Diffraction data were collected at the Australian Synchrotron MX2 beamline (100 K, 13 keV) using an EIGER X 16 M detector. Complete datasets were obtained over 360° with 1° oscillation steps and 1 s exposures. Data were integrated with XDS^56^ and scaled with AIMLESS^57^. Phasing was performed by molecular replacement in Phaser using native DsbA (PDB: 1FVK^26^) as the search model. Model building and refinement were carried out in Coot^58^ and phenix.refine^59^. Model quality was assessed using Rfree values (5% test set) and validated with MolProbity^60^. Structural figures were prepared in PyMOL^61^.

### Structural comparisons, structural motif search and curation

DsbA-like structures (EcDsbA PDB: 1FVK^26^, NmDsbA3 PDB: 3DVX^62^, NmDsbA1 PDB: 3DVW^62^, SeDsbL (PDB: 3L9U^63^), VfDsbA PDB: 3FEU, LpDsbA PDB: 4JRR, CtDsbA PDB: 5KBC^64^, MtbDsbA PDB: 4K6X^65^, PaDsbA1 PDB: 3H93^66^), were retrieved from the PDB. When multiple chains were present, a single representative chain was analysed. Structural superpositions were performed in PyMOL^61^.

A structure-guided search for TRX-fold complexes sharing the *cis-proline* anchoring geometry was performed with FoldDisco^6^. The query motif was built from the DsbA-LptD complex, backbone atoms of DsbA residues V150–*cis*P151 and C30 together with the substrate cysteine from the LptD peptide. Only backbone atoms were constrained to prioritise geometry over sequence.

The resulting search returns from FoldDisco were then limited to the Protein Data Bank deposited structures (PDB; search date: 13.07.2025) with default parameters. Hits were curated by restricting the RMSD values to <0.8 Å and manually searching for complex structures. The final non-redundant set comprised 12 TRX-like enzyme–substrate complexes spanning bacteria, yeast, mammals, plants, and parasites (Supplementary Table 1).

### Statistics and reproducibility

No data were excluded from the analyses. Unless stated otherwise, experiments were repeated in *n* = 3 biologically independent experiments, defined as independent assay runs performed on separate days using independently prepared protein samples. Technical replicates (e.g., replicate wells or repeated measurements within a run) were used to assess measurement precision and were not treated as independent biological replicates. Data are presented as mean ± SEM as indicated. Redox titration data were fit in GraphPad Prism v10 using a two-electron Nernst-based equilibrium model to obtain Keq and redox potentials. For stopped-flow experiments, initial rates were determined from the linear region of the fluorescence traces using Pro-Data SX software, and second-order rate constants were calculated under pseudo-second-order conditions.

### Reporting summary

Further information on research design is available in the Nature Portfolio Reporting Summary linked to this article.

## Supplementary information

Supplementary Information

Description of Additional Supplementary Files

Supplementary Data 1

Supplementary Movie 1.

Supplementary Movie 2.

Reporting Summary

Transparent Peer Review file

## Data Availability

All crystallographic data have been deposited in the Protein Data Bank under accession codes 9Y0O, 9Y0N, 9Y0M, 9Y0P, and 9Y0Q. Numerical source data for graphs can be found in Supplementary Data 1. Uncropped SDS–PAGE gel images are provided in Supplementary Fig. 3. All other data are available from the corresponding author (or other sources, as applicable) on reasonable request.
