# Supplementary material for: A universal cis-proline lock defines catalysis in thioredoxin-fold enzymes
Source: Commun Biol. 2026 Apr 14;9:821. doi: 10.1038/s42003-026-10010-8 (PMC13269488; doi:10.1038/s42003-026-10010-8)
Supplement: Supplementary file 1 — Supplementary Information [file 42003_2026_10010_MOESM1_ESM.pdf]

A universal *cis*-proline lock defines catalysis across thioredoxin-fold enzymes

Taylor Cunliffe[1], Geqing Wang[1], Stephanie Penning[1], Pramod Subedi[1], Makrina Totsika[2], Jason J Paxman[1], Begoña Heras[1]

Supplementary material

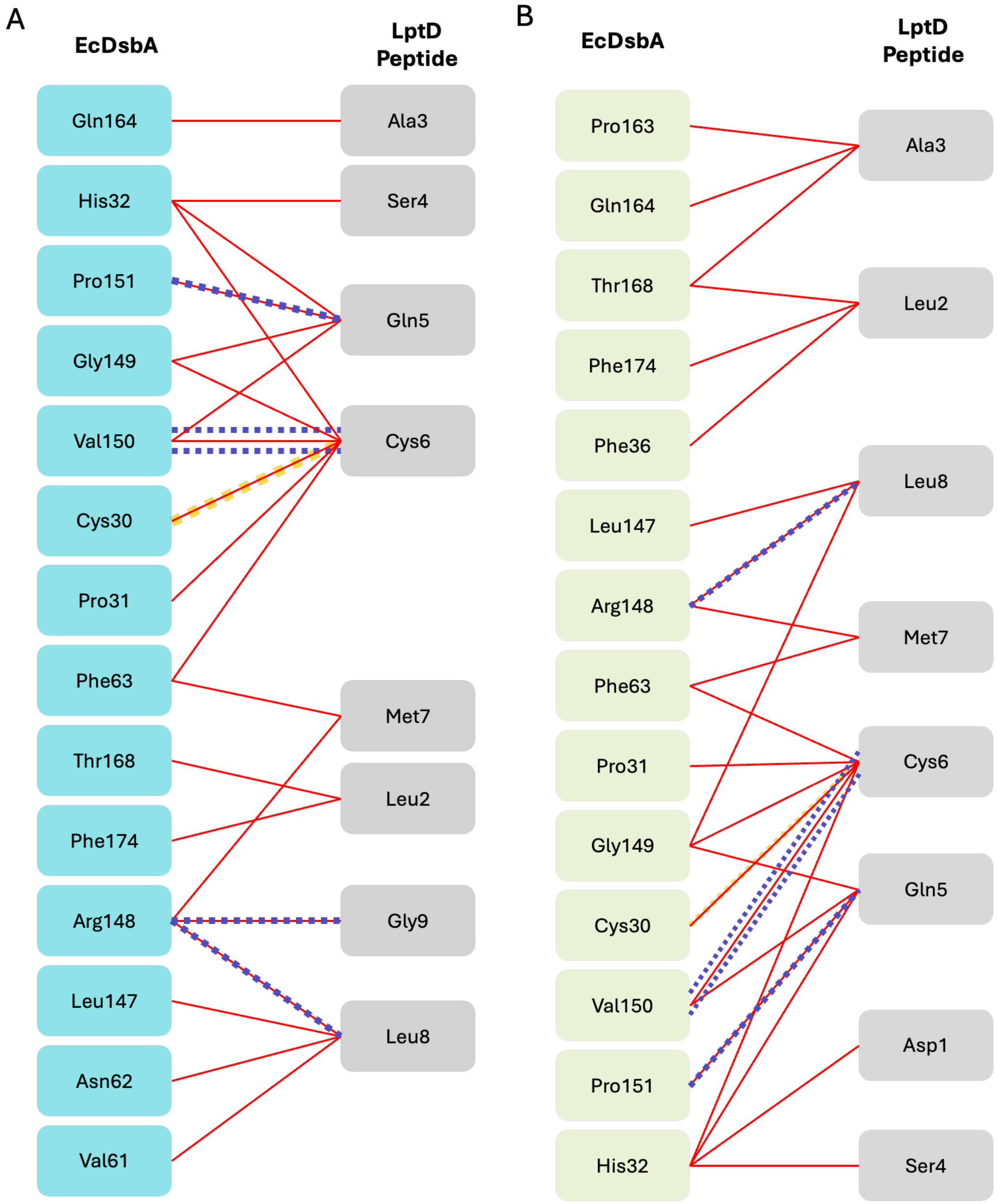

Supplementary Figure 1. *E. coli* DsbA–LptD peptide interaction map. Interaction maps illustrating contacts between DsbA and the LptD-derived peptide in binding mode I (***A***) and binding mode II (***B***). Red lines indicate van der Waals interactions, blue lines represent hydrogen bonds, and yellow lines denote disulfide bond formation between DsbA and the peptide. Interaction maps were generated using PDBsum[1].

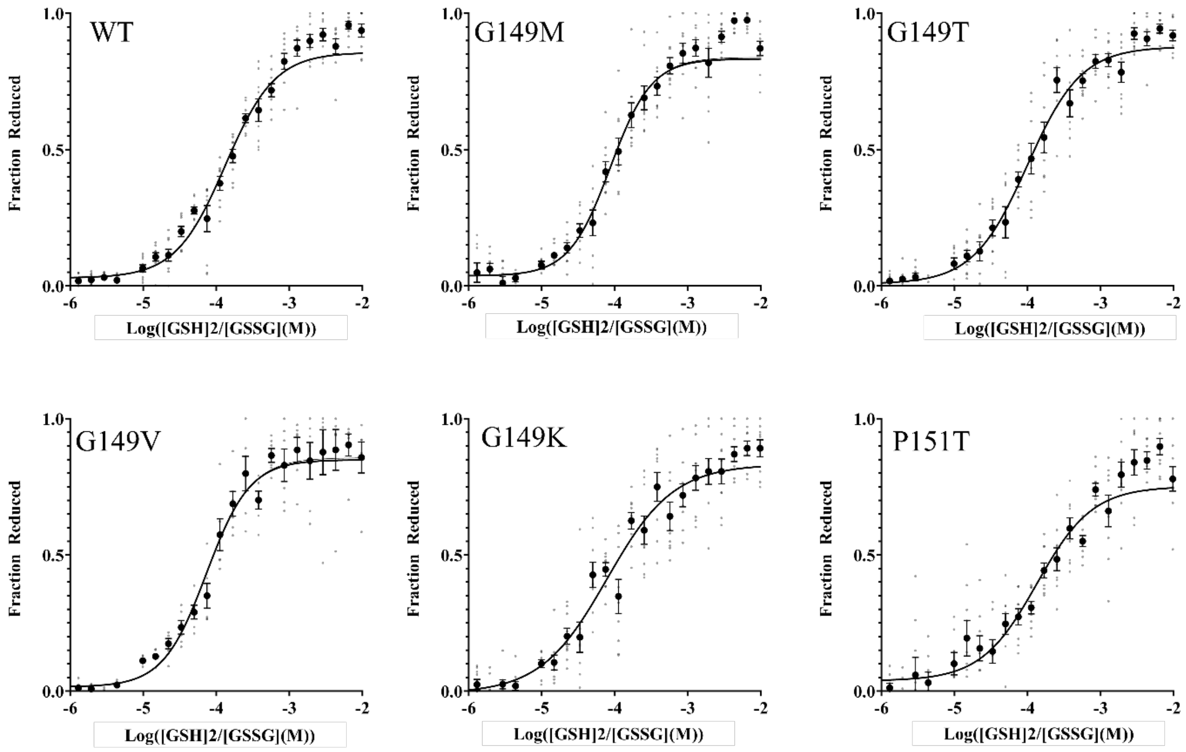

Supplementary Figure 2. Redox potentials of DsbA active-site mutants. Redox potentials of wild-type DsbA and active-site variants shown as mean ± SEM. Measurements were performed in triplicate on three independent occasions.

A

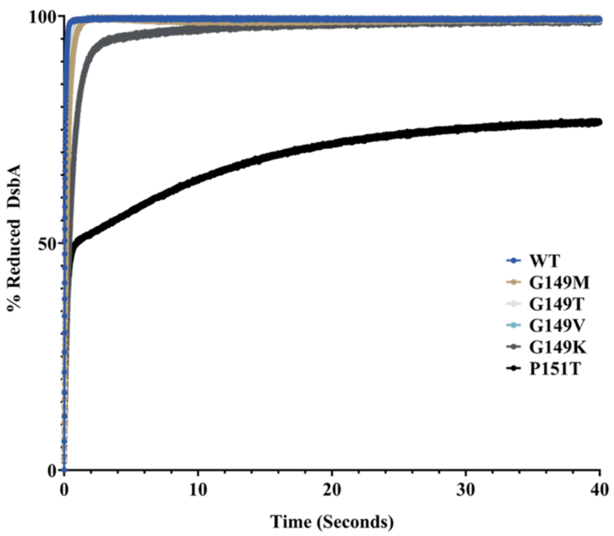

B

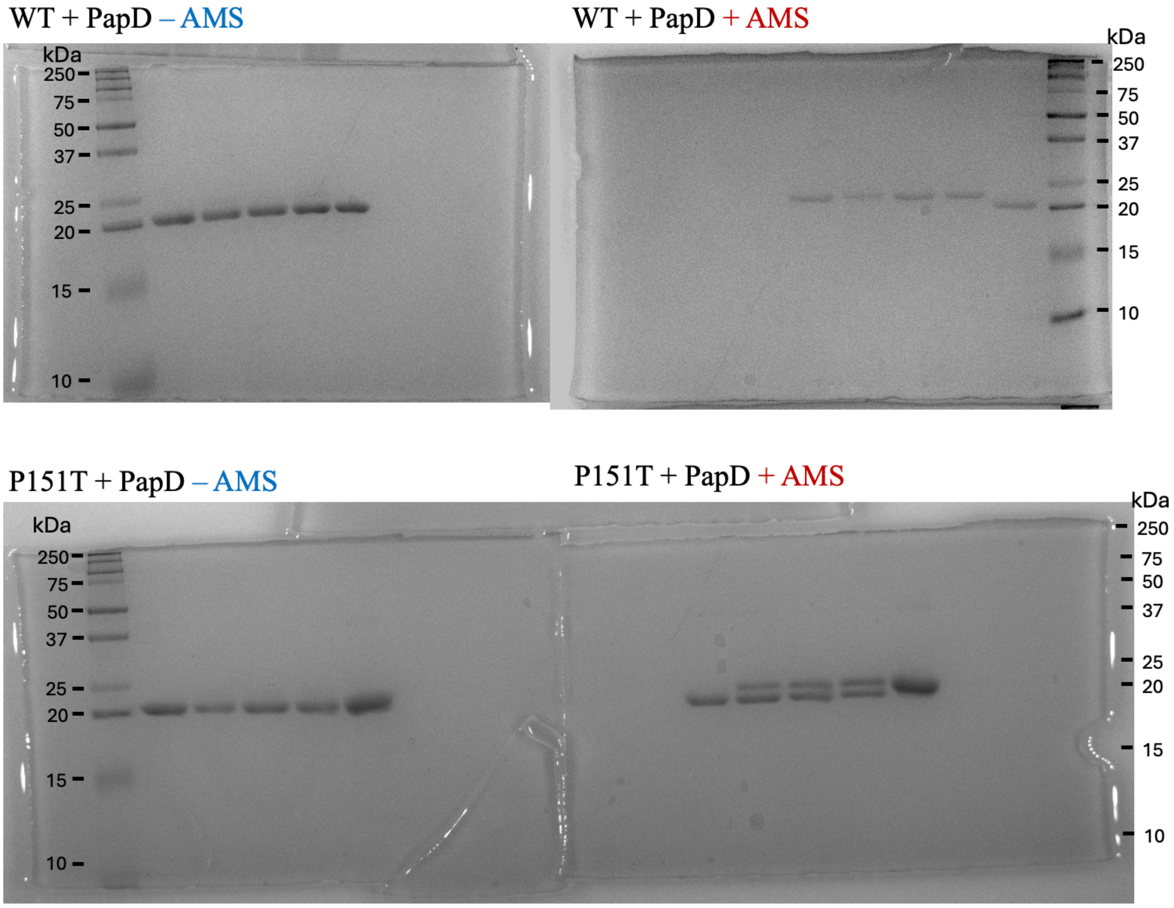

Supplementary Figure 3. Kinetic and biochemical characterisation of DsbA *cis*-proline loop variants. (*A*) Stopped-flow fluorescence traces showing reduction of DsbA *cis*-proline loop mutants over a 40-s time course. Wild-type DsbA and G149 variants (G149M, G149T, G149V, G149K) rapidly approach completion of reduction, whereas the P151T mutant displays a markedly slower reaction and incomplete reduction within the measured time frame.

(***B***) Full, uncropped SDS–PAGE gels corresponding to the electron-transfer assays shown in Fig. 5D. Wild-type DsbA and the P151T mutant were incubated with the PapD peptide substrate in the presence or absence of AMS, as indicated. Molecular weight markers (kDa) are shown, and gels are labelled.

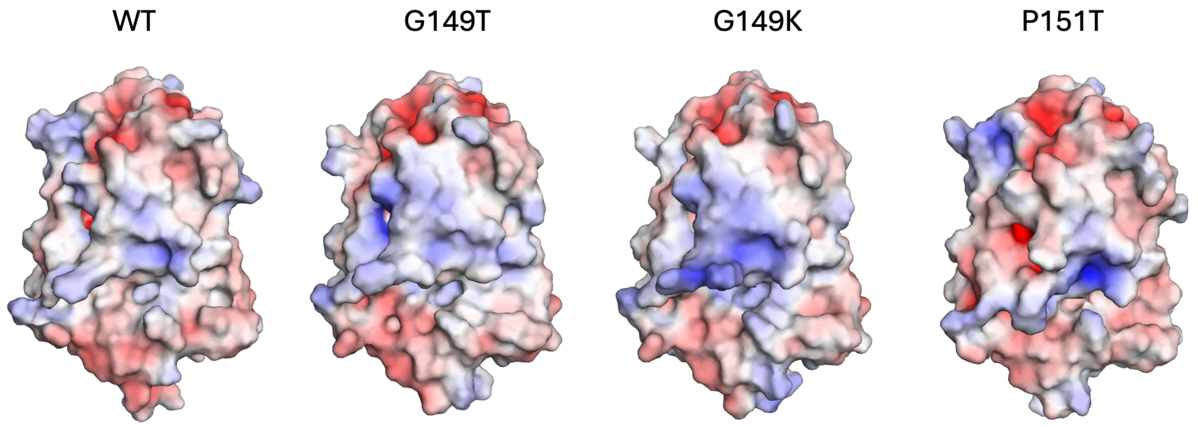

Supplementary Figure 4. Electrostatic surface representations of wild-type and mutant DsbA proteins. Electrostatic surface potentials were calculated using APBS. Positively charged regions are shown in blue (+5 kT/e saturation), and negatively charged regions are shown in red (−5 kT/e saturation). The catalytic face of wild-type DsbA is predominantly hydrophobic, with limited surface charge. Introduction of the G149T mutation results in a modest increase in positive electrostatic potential across the catalytic face, whereas the G149K mutation leads to a pronounced increase in positive charge in proximity to the active site. The P151T mutation also results in conformational changes that expose positive electrostatic potential within the active-site region.

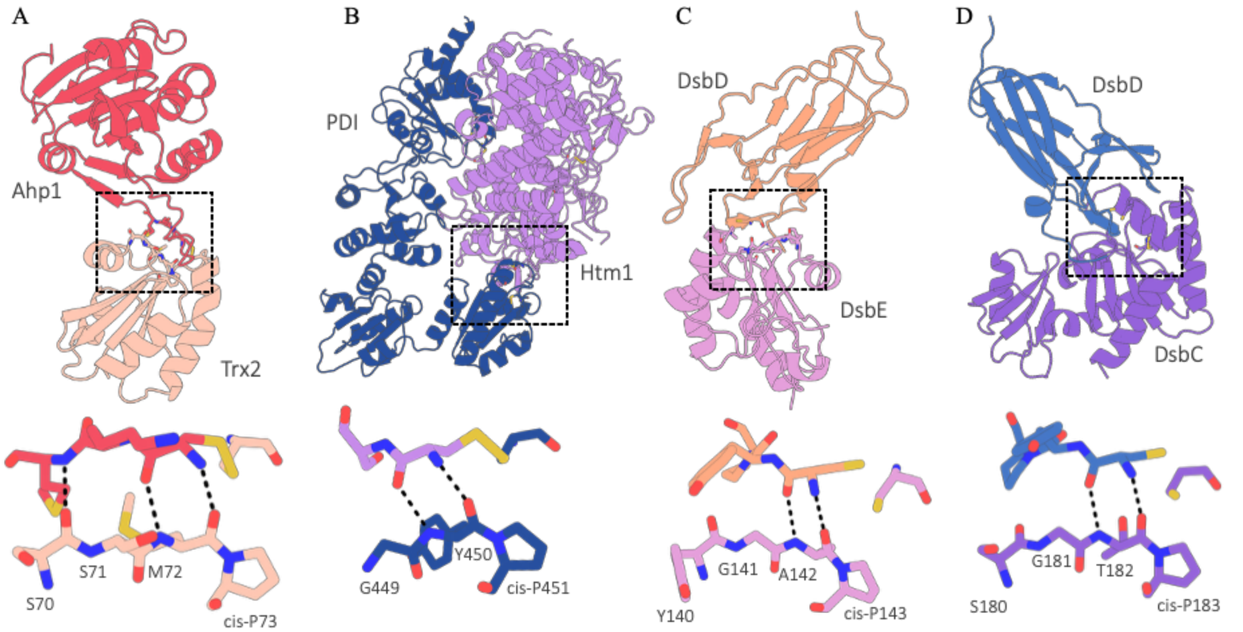

Supplementary Figure 5. Conservation of the *cis*-proline lock across thioredoxin-fold complexes.

(A–D) Representative thioredoxin-fold enzyme complexes from yeast and bacteria illustrate conservation of the *cis*-proline lock adjacent to the catalytic CXXC motif. *(A) Saccharomyces cerevisiae* peroxiredoxin Ahp1 with Trx2 , PDB: 4DSS[2]; *(B) Saccharomyces cerevisiae* protein disulfide isomerase (PDI) with mannosidase Htm1, PDB: 8ZPW[3])*;(C) E. coli* N-terminal DsbD with DsbE, PDB: 1Z5Y[4]; *(D) E. coli* DsbC with N-terminal cDsbD, PDB: 1JZD [5]. Insets show the *cis*-proline loop and surrounding residues, highlighting a conserved hydrogen-bonding geometry that positions the active-site cysteines. Despite substantial divergence in sequence and binding interfaces, the curvature and anchoring interactions of the *cis*-proline loop are preserved, underscoring the conserved catalytic role of this structural motif.

Supplementary Table 1. List of TRX like protein complexes. Protein complexes with similar architectures were identified using a FoldDisco search of the LptD peptide complex structure *cis*-proline residues (R148, G149, V150, cisP151, C30) and the cysteine of the substrate peptide[6].

PDB	Complex	Organism	*Cis-proline* loop composition	Number of hydrogen bonds formed	Reference
4DSS	Ahp/Trx2	*Saccharomyces cerevisiae*	S71, S72, M73, cis-P74	3 (cisP-1 O--N, cisP-1 N--O, cisP-2 O--N)	2
3PIN	Mxr1/Trx2	*Saccharomyces cerevisiae*	S71, S72, M73, cis-P74	3 (cisP-1 O--N, cisP-1 N--O, cisP-2 O--N)	7
3W8J	Pdi1/Prx4 peptide	*Homo sapiens, Mus musculus*	Q96, G97, F98, cis-P99	3 (cisP-1 O--N, cisP-1 N--O, cisP-2 O--N)	8
3WGX	Trx2/Prx4	*Homo sapiens, Mus musculus*	R260, G261,Y262, cis-P263	3 (cisP-1 O--N, cisP-1 N--O, cisP-2 O--N)	8
8ZPW	*Htm1/Pdi1*	*Saccharomyces cerevisiae*	a domain: P103, G104, F105, cisP106 a' domain: E448, G449, Y450, cisP451	2 (cisP-1 O--N, cisP-1 N--O)	3
1JZD	*DsbC/N-terminal DsbD*	*Escherichia coli*	S180, G181, T182, cis-P183	2 (cisP-1 O--N, cisP-1 N--O)	5
1VRS	*N-terminal DsbD/C-terminal DsbD*	*Escherichia coli*	L508, G509, L510, cis-P511	2 (cisP-1 O--N, cisP-1 N--O)	9
4TXV	*Thioredoxin like TlpA/CoxB PD*	*Bradyrhizobium diazoefficiens*	L177, G178, M179, cis-P180	2 (cisP-1 O--N, cisP-1 N--O)	10

1Z5Y	N-Terminal DsbD/DsbE	*Escherichia coli*	Y141, G142, A143, cis-P144	2 (cisP-1 O--N, cisP-1 N--O)	[4]
6YEV	MsrA/Trx	*Escherichia coli*	R73, G74, I75, cis-P76	2 (cisP-1 O--N, cisP-1 N--O)	No reference
4J56	Trx reductase2/Trx	*Plasmodium falciparum*	T70, S71, M72, cis-P73	2 (cisP-1 O--N, cisP-1 N--O)	[11]
2O8V	PAPS reductase/ Trx1	*Escherichia coli*	R73, G74, I75, cis-P76	2 (cisP-1 O--N, cisP-2 O--N)	[12]

Supplementary Table 2. Primers for mutagenesis of *E. coli* DsbA.

**Construct**	**Sequence (5′→3′)**
EcDsbA_G149V_for	TTG CGT GTC GTT CCG GCG ATG TTT G
EcDsbA_G149V_rev	CGG AAC GAC ACG CAA CTG CAC GTC AGC
EcDsbA_G149T_for	TTG CGT ACC GTT CCG GCG ATG TTT G
EcDsbA_G149T_rev	CGG AAC GGT ACG CAA CTG CAC GTC AGC
EcDsbA_G149M_for	TTG CGT ATG GTT CCG GCG ATG TTT G
EcDsbA_G149M_rev	CGG AAC CAT ACG CAA CTG CAC GTC AGC
EcDsbA_G149K_for	TTG CGT AAA GTT CCG GCG ATG TTT GTT AAC
EcDsbA_G149K_rev	CGG AAC TTT ACG CAA CTG CAC GTC AG
EcDsbA_P151T_for	GCG TGG CGT TAC GGC GAT GTT TGT TAA C
EcDsbA_P151T_rev	ATC GCC GTA ACG CCA CGC AAC TGC ACG

Supplementary Table 3. Crystallisation conditions for DsbA constructs in this study.

**Construct**	**Concentration (mg/ml)**	**Condition**
G149T	10	0.2 M Ammonium sulphate, 30% w/v Polyethylene glycol 4,000
G149K	10	0.1 M Sodium acetate trihydrate pH 4.6, 2.0M sodium chloride
P151T	3	1 M Ammonium sulphate and 25% PEG 8000
DsbA-LptD Crystal form I	10	0.2 M Calcium acetate hydrate, 20% w/v Polyethylene glycol 3,350
DsbA-LptD Crystal form II	10	0.2 M Magnesium nitrate hexahydrate, 20% w/v Polyethylene glycol 3,350

Supplementary References

1. Laskowski, R.A., Jabłońska, J., Pravda, L., Vařeková, R.S. & Thornton, J.M. PDBsum: Structural summaries of PDB entries. *Protein Sci.* **27**, 129-134 (2018).
2. Lian, F.-M. et al. Structural Snapshots of Yeast Alkyl Hydroperoxide Reductase Ahp1 Peroxiredoxin Reveal a Novel Two-cysteine Mechanism of Electron Transfer to Eliminate Reactive Oxygen Species. *J.Biol. Chem.* **287**, 17077-17087 (2012).
3. Zhao, D., Wu, X. & Rapoport, T.A. Initiation of ERAD by the bifunctional complex of Mnl1 mannosidase and protein disulfide isomerase. *bioRxiv* 2024.10.17.618908 (2024).
4. Stirnimann, C.U. et al. Structural Basis and Kinetics of DsbD-Dependent Cytochrome c Maturation. *Structure* **13**, 985-993 (2005).
5. Haebel, P.W., Goldstone, D., Katzen, F., Beckwith, J. & Metcalf, P. The disulfide bond isomerase DsbC is activated by an immunoglobulin-fold thiol oxidoreductase: crystal structure of the DsbC–DsbDα complex. *EMBO J.* **21**, 4774-4784 (2002).
6. Kim, H., Kim, R.S., Mirdita, M. & Steinegger, M. Structural motif search across the protein-universe with FoldDisco. *bioRxiv* 2025.07.06.663357 (2025).
7. Ma, X.-X. et al. Structural Plasticity of the Thioredoxin Recognition Site of Yeast Methionine S-Sulfoxide Reductase Mxr1. *J. Biol. Chem.* **286**, 13430-13437 (2011).
8. Sato, Y. et al. Synergistic cooperation of PDI family members in peroxiredoxin 4-driven oxidative protein folding. *Sci. Rep.* **3**, 2456 (2013).
9. Rozhkova, A. et al. Structural basis and kinetics of inter- and intramolecular disulfide exchange in the redox catalyst DsbD. *EMBO J.* **23**, 1709-19 (2004).
10. Abicht, H.K. et al. How Periplasmic Thioredoxin TlpA Reduces Bacterial Copper Chaperone ScoI and Cytochrome Oxidase Subunit II (CoxB) Prior to Metallation. *J. Biol. Chem.* **289**, 32431-32444 (2014).
11. Fritz-Wolf, K. et al. Crystal Structure of the Plasmodium falciparum Thioredoxin Reductase–Thioredoxin Complex. *J. Mol. Biol.* **425**, 3446-3460 (2013).
12. Chartron, J., Shiau, C., Stout, C.D. & Carroll, K.S. 3'-Phosphoadenosine-5'-phosphosulfate Reductase in Complex with Thioredoxin: A Structural Snapshot in the Catalytic Cycle. *Biochemistry* **46**, 3942-3951 (2007).
