# Supplementary material for: A universal cis-proline lock defines catalysis in thioredoxin-fold enzymes
Source: Commun Biol. 2026 Apr 14;9:821. doi: 10.1038/s42003-026-10010-8 (PMC13269488; doi:10.1038/s42003-026-10010-8)
Supplement: Supplementary file 2 — Description of Additional Supplementary Files [file 42003_2026_10010_MOESM2_ESM.docx]

**Description of Additional Supplementary Files**

**File name:** Supplementary Data 1

**Description:** Numerical source data for graphs and charts in the paper and supplementary material.

**File name:** Supplementary Movie 1 - Loop 1 mobility in DsbA during LptD peptide engagement.

**Description:** PyMOL (Rigimol) morph interpolating between apo DsbA and two DsbA–LptD mixeddisulfide structures (binding mode I and II, green). Loop 1 (F63–G66) is highlighted, showing its outward displacement that enlarges the catalytic groove to accommodate the LptD peptide (white), while the Cys30– Pro31–His32–Cys33 motif and cisPro151 remain constrained.

**File name:** Supplementary Movie 2 - Conformational flexibility of the cis-proline loop induced by the P151T mutation.

**Description:** PyMOL (Rigimol) morph interpolating between the four protomers in the asymmetric unit of the DsbA P151T crystal structure. The substitution of cisPro151 with threonine converts the peptide bond from cis to trans, resulting in increased loop mobility and heterogeneity in the positioning of the V150 carbonyl adjacent to the catalytic site.
