# Supplementary material for: A universal cis-proline lock defines catalysis in thioredoxin-fold enzymes
Source: Commun Biol. 2026 Apr 14;9:821. doi: 10.1038/s42003-026-10010-8 (PMC13269488; doi:10.1038/s42003-026-10010-8)
Supplement: Supplementary file 6 — Reporting Summary [file 42003_2026_10010_MOESM6_ESM.pdf]

nature portfolio

Corresponding author(s): Begona Heras

Last updated by author(s): Mar 2, 2026

Reporting Summary

Nature Portfolio wishes to improve the reproducibility of the work that we publish. This form provides structure for consistency and transparency in reporting. For further information on Nature Portfolio policies, see our Editorial Policies and the Editorial Policy Checklist.

Statistics

For all statistical analyses, confirm that the following items are present in the figure legend, table legend, main text, or Methods section.

n/a	Confirmed	
☐	☒	The exact sample size (*n*) for each experimental group/condition, given as a discrete number and unit of measurement
☐	☒	A statement on whether measurements were taken from distinct samples or whether the same sample was measured repeatedly
☐	☒	The statistical test(s) used AND whether they are one- or two-sided<br>*Only common tests should be described solely by name; describe more complex techniques in the Methods section.*
☐	☒	A description of all covariates tested
☒	☐	A description of any assumptions or corrections, such as tests of normality and adjustment for multiple comparisons
☐	☒	A full description of the statistical parameters including central tendency (e.g. means) or other basic estimates (e.g. regression coefficient) AND variation (e.g. standard deviation) or associated estimates of uncertainty (e.g. confidence intervals)
☐	☒	For null hypothesis testing, the test statistic (e.g. *F*, *t*, *r*) with confidence intervals, effect sizes, degrees of freedom and *P* value noted<br>*Give P values as exact values whenever suitable.*
☒	☐	For Bayesian analysis, information on the choice of priors and Markov chain Monte Carlo settings
☒	☐	For hierarchical and complex designs, identification of the appropriate level for tests and full reporting of outcomes
☒	☐	Estimates of effect sizes (e.g. Cohen's *d*, Pearson's *r*), indicating how they were calculated

Our web collection on statistics for biologists contains articles on many of the points above.

Software and code

Policy information about availability of computer code

Data collection

X-ray diffraction data were collected at the Australian Synchrotron on the MX2 Beamline (Aragao, D. et al. Journal of Synchrotron Radiation 25, 885-891, d(2018); using the Blue Ice software (McPhillips et al. J. Synchrotron Rad. (2002). 9, 401-406) and the QEGui Eiger graphics interphase.

Data analysis

Protein structure determination by X-ray crystallography

- The X-ray diffraction data was indexed and integrated using XDS (Kabsch, W. Acta Crystallographica Section D 66, 133-144, (2010)) and scaled using AIMLESS (Evans PR, Murshudov GN. Acta Crystallographica Section D: Biological Crystallography. 2013;69:1204-14)

- The structures were solved using Molecular Replacement phasing protocol in Phenix (Adams, P. D. et al. PHENIX: building new software for automated crystallographic structure determination. Acta Crystallographica Section D: Biological Crystallography 58, 1948-1954 (2002))

- The models were refined against the native dataset using phenix.refine in Phenix and further built using COOT ((Emsley, P. & Cowtan, K. Acta Crystallogr D Biol Crystallogr 60, 2126-32 2004).

- The quality of the models were assessed by MolProbity (Davis, I.W. et al. Nucleic Acids Res 35, W375-83 2007)

- Protein structure figures were created with PyMOL (DeLano, W.L. The PyMOL Molecular Graphics System, http://www.pymol.org/ . DeLano Scientific, San Carlos, CA, USA. 2002

- A structure-guided search for TRX-fold complexes sharing the cis-proline anchoring geometry was performed with FoldDisco Kim, H., Kim, R. S., Mirdita, M. & Steinegger, M. Structural motif search across the protein-universe with Folddisco. bioRxiv, 2025.2007.2006.663357, doi:10.1101/2025.07.06.663357 (2025).

For manuscripts utilizing custom algorithms or software that are central to the research but not yet described in published literature, software must be made available to editors and reviewers. We strongly encourage code deposition in a community repository (e.g. GitHub). See the Nature Portfolio guidelines for submitting code & software for further information.

Data

Policy information about availability of data

All manuscripts must include a data availability statement. This statement should provide the following information, where applicable:

- Accession codes, unique identifiers, or web links for publicly available datasets
- A description of any restrictions on data availability
- For clinical datasets or third party data, please ensure that the statement adheres to our policy

The crystallography, atomic coordinates, and structure factors reported in this paper have been deposited in the Protein Data Bank, www.pdb.org (PDB ID codes 9Y0O 9Y0N 9Y0M 9Y0P 9Y0Q)

Human research participants

Policy information about studies involving human research participants and Sex and Gender in Research.

Reporting on sex and gender	*Use the terms sex (biological attribute) and gender (shaped by social and cultural circumstances) carefully in order to avoid confusing both terms. Indicate if findings apply to only one sex or gender; describe whether sex and gender were considered in study design whether sex and/or gender was determined based on self-reporting or assigned and methods used. Provide in the source data disaggregated sex and gender data where this information has been collected, and consent has been obtained for sharing of individual-level data; provide overall numbers in this Reporting Summary. Please state if this information has not been collected. Report sex- and gender-based analyses where performed, justify reasons for lack of sex- and gender-based analysis.*
Population characteristics	*Describe the covariate-relevant population characteristics of the human research participants (e.g. age, genotypic information, past and current diagnosis and treatment categories). If you filled out the behavioural & social sciences study design questions and have nothing to add here, write "See above."*
Recruitment	*Describe how participants were recruited. Outline any potential self-selection bias or other biases that may be present and how these are likely to impact results.*
Ethics oversight	*Identify the organization(s) that approved the study protocol.*

Note that full information on the approval of the study protocol must also be provided in the manuscript.

Field-specific reporting

Please select the one below that is the best fit for your research. If you are not sure, read the appropriate sections before making your selection.

☒ Life sciences ☐ Behavioural & social sciences ☐ Ecological, evolutionary & environmental sciences

For a reference copy of the document with all sections, see nature.com/documents/nr-reporting-summary-flat.pdf

Life sciences study design

All studies must disclose on these points even when the disclosure is negative.

Sample size	Sample sizes were not predetermined based on statistical methods, but were chosen according to the standards in the field - at least 3 independent biological replicates for each condition. Where appropriate, this generated sufficient data for statistical analysis.
Data exclusions	No data was excluded from this manuscript
Replication	Reported results were consistently replicated across multiple experiments with all replicates generating similar results.
Randomization	No randomization was necessary as experiments were performed with appropriate controls. Randomization is not generally used in this field.
Blinding	Investigators were not blinded. Blinding during analysis was not necessary because the results are quantitative and did not require subjective

Blinding	judgment or interpretation. Blinding is not typically used in the field.

Reporting for specific materials, systems and methods

We require information from authors about some types of materials, experimental systems and methods used in many studies. Here, indicate whether each material, system or method listed is relevant to your study. If you are not sure if a list item applies to your research, read the appropriate section before selecting a response.

Materials & experimental systems

n/a	Involved in the study
☐	☒ Antibodies
☒	☐ Eukaryotic cell lines
☒	☐ Palaeontology and archaeology
☒	☐ Animals and other organisms
☒	☐ Clinical data
☒	☐ Dual use research of concern

Methods

n/a	Involved in the study
☒	☐ ChIP-seq
☒	☐ Flow cytometry
☒	☐ MRI-based neuroimaging

Antibodies

Antibodies used	N.A.
Validation	N.A.
