# Supplementary material for: A universal cis-proline lock defines catalysis in thioredoxin-fold enzymes
Source: Commun Biol. 2026 Apr 14;9:821. doi: 10.1038/s42003-026-10010-8 (PMC13269488; doi:10.1038/s42003-026-10010-8)
Supplement: Supplementary file 7 — Transparent Peer Review file [file 42003_2026_10010_MOESM7_ESM.pdf]

nature portfolio

Peer Review File

A universal cis-proline lock defines catalysis in thioredoxin-fold enzymes

Corresponding Author: Professor Begona Heras

This manuscript has been previously submitted at another journal. This document only contains information relating to versions considered at Communications Biology.

This file contains all reviewer reports in order by version, followed by all author rebuttals in order by version.

Version 0:

Reviewer comments:

Reviewer #1

(Remarks to the Author)
This manuscript investigates the role of the conserved cis-proline residue in the disulfide oxidase DsbA, a member of thioredoxin-fold enzyme family. The authors conducted structural and biochemical analyses, including the structural determination of a covalent complex between DsbA and a peptide derived from LptD. The study provides important mechanistic insights into the role of the cis-proline loop in DsbA catalysis. A previous structural study (ref. 40) reported a DsbA–SigA peptide complex in which the peptide was covalently linked through a thioether bond rather than a native disulfide bond. In contrast, the present manuscript describes the structure of a more physiologically relevant complex. Given that the cis-proline loop is conserved among thioredoxin-fold proteins, this work would be of interests to enzymatic community. However, there are several issues that should be addressed in the revised manuscript.

1. lines 78–80: The Introduction section would be improved with a more detailed explanation of the DsbA catalytic mechanism. In particular, the roles of Cys30 and Cys33 should be described. This would help readers understand how DsbA introduces disulfide bonds into various proteins.

2. lines 97-99: Although the Methods section explains the strategy used to obtain the covalent protein–peptide complex (e.g., use of the C33A mutant), this information should also be described briefly in the Results section (lines 97–99). Currently, it is not clear to readers why the C33A mutant was used and how the covalent intermediate was stabilized, because the catalytic mechanism of DsbA is not described in the manuscript. In addition, the difference between this strategy and the one used in ref. 40 could be clarified.

3. lines 138-140: In the presented DsbA structure with the LptD peptide, the peptide substrate interacts with the hydrophobic groove. However, the functional role of this hydrophobic groove is not explained in the manuscript. The authors should discuss its functional importance in the DsbA reaction.

4. Line 149：“?” should be removed.

5. Fig. 1E：The region colored in black is not explained in the figure legend. The authors should include a sentence of what this region represents in the figure legend.

6. Fig. 4A–B: It is difficult to distinguish individual lines due to the grayscale representation. The authors should consider using color to enhance visibility.

7. Fig. 4D: The authors should include an assay using the wild-type DsbA as a control.

Reviewer #2

(Remarks to the Author)
This manuscript by Cunliffe et al. presents a compelling and carefully executed study that addresses an important

mechanistic question in thioredoxin fold enzymology, namely how these enzymes maintain catalytic precision while acting on chemically diverse substrates. The combination of structural, biochemical, and kinetic evidence is strong, and the identification of a conserved cis proline loop as a unifying catalytic element is both elegant and convincing. I find the work solid and very interesting. The following comments are intended to improve clarity, contextualisation, and overall interpretability of the manuscript.

1. Introductory figure. I recommend adding a dedicated introductory figure that presents both a schematic of the thiol-disulfide exchange reaction and a simplified depiction of a thioredoxin fold, ideally using DsbA as the example and clearly labelling the catalytic motif, the cis proline loop, and the surrounding structural elements. This material appears later in the manuscript (Fig. 1AB), I would move it to an early, self-contained overview figure as this would greatly assist readers who are less familiar with the system and provide a clearer conceptual foundation for the mechanistic model that follows.

2. Lines 134 to 143, peptide mimics versus natural substrates. In the discussion comparing natural substrates with peptide mimics, it would be helpful to acknowledge more explicitly that peptide-based systems cannot be assumed to capture the full complexity of protein-protein interactions. The manuscript itself shows that peptide derived interactions can differ substantially from those observed or expected for full length protein substrates, and these differences often appear to correlate with substrate type, namely peptides versus intact proteins. Given that SigA is currently the only natural substrate for which a structure is available, it remains uncertain how faithfully peptide mimics reproduce native binding modes more broadly. It is worth noting that the interaction with DsbB appears consistent between a peptide mimic and the full-length protein, although this case is unusual because the redox state of DsbA is reversed. A brief clarification of these points in this section would help avoid overinterpretation of peptide-based binding modes and ensure that their limitations are clearly framed.

3. Lines 155 to 169, consensus sequence for the cis proline loop. Given the clear sequence trends highlighted for the cis proline loop, it would strengthen the manuscript to propose a concise consensus of these sequence features (maybe a motif logo would be useful here).

4. Figure 4D, inclusion of wild type DsbA. The assays presented in Figure 4D would be much clearer if wild-type DsbA were shown directly alongside the P151T variant. This applies both to the non-AMS gel, where the absence of trapped intermediates in wild-type should be expected under identical conditions, and to the AMS labelled gel, where the redox state of wild type would serve as an essential comparison for the partially oxidised P151T sample.

5. Lines 180 to 183, G149M and G149T variants. The discussion of G149 variants in this section is currently somewhat confusing. G149M retains near wild-type activity despite introducing a relatively bulky residue, whereas G149T, which is described earlier as an acceptable amino acid at this position, has measurably reduced activity. Readers may struggle to understand this apparent inconsistency. Expanding the explanation to address how differences in side chain chemistry, polarity, or microenvironmental effects can influence catalytic turnover, even in the absence of major structural perturbation, would help reconcile these observations.

6. Lines 213 to 214, explanation for reduced efficiency of G149T. The statement that G149T leaves the local environment largely unchanged while still exhibiting reduced catalytic efficiency may be puzzling to readers. It would be helpful to elaborate that catalytic efficiency in thioredoxin fold enzymes can depend sensitively on dynamic or microenvironmental factors, such as local hydration, side chain orientation, or slight shifts in conformational sampling. These factors may not significantly affect the redox potential or the static structure, yet they can influence the rate of mixed disulfide formation or resolution. A brief clarification here would strengthen the mechanistic interpretation.

Thank you for the opportunity to review this really nice paper!

Version 1:

Reviewer comments:

Reviewer #1

(Remarks to the Author)
The authors have responded appropriately to the comments from the reviewers.

Reviewer #2

(Remarks to the Author)
In the revised version of this manuscript the authors have addressed fully all of my previous comments and concerns. I commend them for doing so thoroughly and thoughtfully. I am happy to recommend this manuscript for publication.

Open Access This Peer Review File is licensed under a Creative Commons Attribution 4.0 International License, which permits use, sharing, adaptation, distribution and reproduction in any medium or format, as long as you give appropriate credit to the original author(s) and the source, provide a link to the Creative Commons license, and indicate if changes were made.
In cases where reviewers are anonymous, credit should be given to 'Anonymous Referee' and the source.
The images or other third party material in this Peer Review File are included in the article's Creative Commons license, unless indicated otherwise in a credit line to the material. If material is not included in the article's Creative Commons license and your intended use is not permitted by statutory regulation or exceeds the permitted use, you will need to obtain permission directly from the copyright holder.
To view a copy of this license, visit https://creativecommons.org/licenses/by/4.0/

Response to the Reviewers

We thank both reviewers for their evaluation of our manuscript and their constructive comments. We are pleased that they find the work mechanistically compelling and of broad interest to the thioredoxin/oxidoreductase community. Below, we address each comment point by point. All changes are indicated in the revised manuscript.

Referee #1

We thank the reviewer for recognising the mechanistic significance of our study and for highlighting the value of capturing a physiologically relevant DsbA–substrate intermediates. We have addressed all points raised, which have improved clarity and presentation.

1. *Lines 78–80: The Introduction section would be improved with a more detailed explanation of the DsbA catalytic mechanism. In particular, the roles of Cys30 and Cys33 should be described. This would help readers understand how DsbA introduces disulfide bonds into various proteins.*

Response:
We have expanded the Introduction to explicitly describe the sequential roles of Cys30 and Cys33 in thiol–disulfide exchange. Specifically the text reads (lines 79-87) :

...mechanism of DsbA mediated disulfide exchange is well established. In its oxidised state, DsbA presents an active-site Cys30–Cys33 disulfide that functions as the immediate disulfide donor to substrate proteins (Fig. 1C). Catalysis proceeds via nucleophilic substitution, in which a reduced substrate cysteine attacks Cys30, cleaving the DsbA active-site disulfide and forming a transient mixed-disulfide intermediate. Resolution by a second substrate cysteine yields an intramolecular disulfide in the folding protein and leaves DsbA reduced. Cys30 is the cysteine that directly engages the substrate and is intrinsically highly reactive, whereas Cys33 serves as the partner cysteine of the active-site disulfide, contributing to efficient resolution and recycling of the catalytic motif[31-37]. Despite this well-defined chemical mechanism, the structural basis by which DsbA, and TRX-fold enzymes more broadly, accommodate and efficiently catalyse thiol-disulfide exchange reactions in diverse substrates remains unknown.

This addition provides necessary mechanistic context for readers less familiar with DsbA catalysis.

2. Lines 97-99: Although the Methods section explains the strategy used to obtain the covalent protein–peptide complex (e.g., use of the C33A mutant), this information should also be described briefly in the Results section (lines 97–99). Currently, it is not clear to readers why the C33A mutant was used and how the covalent intermediate was stabilized, because the catalytic mechanism of DsbA is not described in the manuscript. In addition,

the difference between this strategy and the one used in ref. 40 could be clarified. Clarification of the use of the C33A mutant and comparison to ref. 40 (lines 97–99)

Response:
We thank the reviewer for pointing out this gap in the Results section. We have now added a brief explanation explaining why the C33A mutant was used, namely to stabilise the mixed-disulfide intermediate by preventing its resolution, thereby enabling structural capture. We also explicitly contrast this strategy with that used in ref. 40, where a thioether-linked peptide was employed to irreversibly trap the complex. This clarification highlights why the present structure more closely represents a native catalytic intermediate. The text now reads as follows (modifications shown in blue, lines 111-117):

To understand how thioredoxin-fold enzymes engage their substrates, we solved crystal structures of E. coli DsbA covalently linked to a peptide from the essential outer membrane protein LptD[9,12,38,39]. Because DsbA rapidly resolves enzyme–substrate disulfide intermediates during catalysis, we stabilised this transient species by substituting the C-terminal active-site cysteine (Cys33) with alanine. The resulting C33A variant permits formation of the mixed disulfide via Cys30 but prevents its resolution, enabling isolation of a covalent DsbA–substrate complex for structural analysis. This strategy differs from that used previously[40], which employed an irreversible thioether linkage to trap a substrate mimic rather than the native mixed-disulfide intermediate.

3. Lines 138-140: In the presented DsbA structure with the LptD peptide, the peptide substrate interacts with the hydrophobic groove. However, the functional role of this hydrophobic groove is not explained in the manuscript. The authors should discuss its functional importance in the DsbA reaction.

Response:
We agree that the functional significance of the hydrophobic groove should be made explicit. We have expanded the section where substrate peptides are first described as binding to this region (lines 121–123) to clarify that the hydrophobic groove functions as a substrate-interaction surface during substrate oxidation and also participates in DsbB interactions during enzyme reoxidation. The text has been modified as follows (modifications shown in blue, lines 121-123):

The resulting structures, determined at 1.47 Å resolution (Table 1), captured two distinct substrate-binding modes (Fig. 2 A, B). In both, the substrate cysteine was covalently attached to the catalytic Cys30 of DsbA, and the peptide's N-terminus extended into the hydrophobic groove of the thioredoxin domain. This groove lies adjacent to the active site (Fig. 1B) and has been implicated in both substrate recognition[21] and DsbB interactions required for DsbA reoxidation[41,42]. However, in these crystal forms the peptide backbone adopted different orientations relative to the active

4. Line 149：“?” should be removed.

Response:
This typographical error has been corrected.

5. Figure 1E: explanation of the black-coloured region

Response:
We have revised the figure legend (new Figure number Fig. 2*C*) to clarify that the black-coloured region corresponds to the bound LptD-derived peptide shown in the overlay (lines 596-597).

6. Fig. 4A–B: It is difficult to distinguish individual lines due to the grayscale representation. The authors should consider using color to enhance visibility.

Response:
Figures 5A, B and C (previously 4A, B, C) have been updated to use colour, with consistent colour coding across panels to clearly distinguish variants.

7. Fig. 4D: The authors should include an assay using the wild-type DsbA as a control.

Response:
We agree that inclusion of wild-type DsbA strengthens the interpretation. We have now added wild-type DsbA controls to both the non-AMS and AMS-labelled gels in Figure 5D top panels (Former Figure 4), allowing direct comparison with the P151T variant (bottom panels) and clearly demonstrating the absence of trapped intermediates and the expected redox behaviour of wild-type enzyme.

Referee #2

We thank the reviewer for their very positive assessment of the study and for the thoughtful suggestions aimed at improving clarity and conceptual framing. We have addressed all points below.

1. Introductory figure. I recommend adding a dedicated introductory figure that presents both a schematic of the thiol-disulfide exchange reaction and a simplified depiction of a thioredoxin fold, ideally using DsbA as the example and clearly labelling the catalytic motif, the cis proline loop, and the surrounding structural elements. This material appears later in the manuscript (Fig. 1AB), I would move it to an early, self-contained overview figure as this would greatly assist readers who are less familiar with the system and provide a clearer conceptual foundation for the mechanistic model that follows.

Response:
We agree that an early overview figure would be highly beneficial. We have therefore added a new introductory figure (new Fig. 1) presenting: (A-B): a simplified representation of the thioredoxin fold using DsbA as an example, with the catalytic CXXC motif, cis-proline loop, and surrounding structural elements clearly labelled (Figure 1A/B in the former version), and

(C): a schematic of DsbA mediated thiol–disulfide exchange and (ii). This new figure 1 is now a the front of the manuscript and provides a conceptual framework for the manuscript.

2. Lines 134 to 143, peptide mimics versus natural substrates. In the discussion comparing natural substrates with peptide mimics, it would be helpful to acknowledge more explicitly that peptide-based systems cannot be assumed to capture the full complexity of protein-protein interactions. The manuscript itself shows that peptide derived interactions can differ substantially from those observed or expected for full length protein substrates, and these differences often appear to correlate with substrate type, namely peptides versus intact proteins. Given that SigA is currently the only natural substrate for which a structure is available, it remains uncertain how faithfully peptide mimics reproduce native binding modes more broadly. It is worth noting that the interaction with DsbB appears consistent between a peptide mimic and the full-length protein, although this case is unusual because the redox state of DsbA is reversed. A brief clarification of these points in this section would help avoid overinterpretation of peptide-based binding modes and ensure that their limitations are clearly framed.

Response: We agree that peptide-based systems should not be assumed to capture the full complexity of interactions formed by intact protein substrates. We have revised this section to clarify that all currently available DsbA–substrate complex structures involve peptide-derived intermediates (lines 159-180), whereas the only structure with a full-length protein partner is the DsbA–DsbB complex (lines 183-186). We also explicitly acknowledge that peptide-derived binding modes may differ from those of intact substrates. At the same time, we clarify that DsbA engages substrates during translocation and early folding, when proteins are unfolded or partially folded, such that peptide substrates provide a relevant approximation of the reactive cysteine-containing segments encountered during catalysis. The text reads as follows (lines 196-203):

While peptide-based substrates cannot be assumed to fully recapitulate the complexity of interactions formed by intact protein substrates, they nevertheless provide valuable mechanistic insight. Indeed, DsbA engages substrates during translocation to the periplasm when proteins are unfolded or partially folded, and recognition is dominated by exposed cysteine-containing segments rather than fully folded domains. In this context, peptide substrates provide a relevant approximation of the reactive state encountered during catalysis, capturing key features of mixed-disulfide formation and cysteine positioning, even if additional contacts contribute to substrate recognition in full-length proteins.

These revisions frame peptide-based structures as mechanistically informative while clearly defining their limitations.

3. Lines 155 to 169, consensus sequence for the cis proline loop. Given the clear sequence trends highlighted for the cis proline loop, it would strengthen the manuscript to propose a concise consensus of these sequence features (maybe a motif logo would be useful here).

Response:
We have revised this section to propose a concise consensus based on our comparisons, highlighting a variable residue at position −3, a small residue at position −2, a compact residue at position −1, and an invariant cis-proline. In addition, we have added a motif logo to visually summarise these trends, which reads as follows (lines 234-237)

Together, these trends define a concise cis-proline loop consensus characterised by a variable residue at position −3, a small residue at position −2, a compact residue at position −1, and an invariant cis-proline that together preserve the backbone geometry required for substrate cysteine anchoring (consensus motif: X–[G/A/S/T]–[V/T]–P(cis)).

4. Figure 4D, inclusion of wild type DsbA. The assays presented in Figure 4D would be much clearer if wild-type DsbA were shown directly alongside the P151T variant. This applies both to the non-AMS gel, where the absence of trapped intermediates in wild-type should be expected under identical conditions, and to the AMS labelled gel, where the redox state of wild type would serve as an essential comparison for the partially oxidised P151T sample.

Response:
As noted above in response to Referee #1, we have now included wild-type DsbA controls in both AMS-labelled and non-labelled gels in Figure 5D. This addition clarifies that the behaviour observed for P151T is distinct from wild type under identical conditions.

5. Lines 180 to 183, G149M and G149T variants. The discussion of G149 variants in this section is currently somewhat confusing. G149M retains near wild-type activity despite introducing a relatively bulky residue, whereas G149T, which is described earlier as an acceptable amino acid at this position, has measurably reduced activity. Readers may struggle to understand this apparent inconsistency. Expanding the explanation to address how differences in side chain chemistry, polarity, or microenvironmental effects can influence catalytic turnover, even in the absence of major structural perturbation, would help reconcile these observations.

Response:
We thank the reviewer for highlighting this apparent inconsistency. We have clarified the text by explicitly distinguishing endpoint turnover from kinetic rate measurements. While G149M, G149V, and G149T support near wild-type turnover in the FRET-based endpoint assay, stopped-flow kinetics reveal that substitutions at position 149 (G149M/V/T) all incur a comparable ~2-fold reduction in the second-order rate constant relative to wild type, despite markedly different side-chain sizes and chemistries. This indicates that side-chain bulk alone is not the primary determinant at this position; rather, substitutions at G149 modulate turnover through local microenvironmental effects (steric, polarity, and electrostatics) that influence transient substrate alignment and mixed-disulfide formation and resolution without causing major structural rearrangements. The revised text now reads as follows (lines 263–274):

Stopped-flow kinetics provided a quantitative framework to interpret these trends. Although G149V, G149T, and G149M all supported near–wild-type turnover in endpoint peptide oxidation assays, kinetic analysis revealed that these variants oxidised a PapD-derived peptide[24] with similar ~2-fold reduced rate constants relative to wild-type DsbA (4.1 × 10^5 $M^{-1}s^{-1}$), clustering within a narrow range (22–52% of wild type) (Fig. 5C, Table 2, Fig. S3). Notably, G149M, G149V, and G149T exhibit comparable kinetic penalties despite markedly different side-chain sizes and chemistries, indicating that side-chain size per se is not the primary determinant of activity at this position. Instead, substitution at G149 modulates catalytic efficiency through chemical and microenvironmental effects, including polarity, hydrophobicity, and charge, that influence substrate binding, mixed-disulfide formation, and resolution. The more pronounced defect observed for G149K is consistent with an additional kinetic penalty imposed by introduction of a charged side chain.

6. Lines 213 to 214, explanation for reduced efficiency of G149T. The statement that G149T leaves the local environment largely unchanged while still exhibiting reduced catalytic efficiency may be puzzling to readers. It would be helpful to elaborate that catalytic efficiency in thioredoxin fold enzymes can depend sensitively on dynamic or microenvironmental factors, such as local hydration, side chain orientation, or slight shifts in conformational sampling. These factors may not significantly affect the redox potential or the static structure, yet they can influence the rate of mixed disulfide formation or resolution. A brief clarification here would strengthen the mechanistic interpretation.

Response:
We agree that this point required clarification. In the revised results, we now make explicit that the reduced catalytic efficiency of G149 variants is not explained by changes in oxidising power or disruption of the cis-proline loop geometry, both of which are preserved in these mutants. Instead, we clarify that substitution at position G149 perturbs the local active-site environment adjacent to the catalytic cysteine, introducing steric and chemical (polarity and charge) changes that can restrict local conformational sampling and may impair productive substrate engagement for the tested peptide substrates. This expanded explanation better reconciles the structural and kinetic observations. The results read as follows (lines 328-345, modification shown in blue).

In contrast, G149T and G149K closely resembled wild-type (RMSD 0.48–0.50 Å over 172 Cα atoms) and preserved the canonical cis-proline loop geometry (Fig. 6E-F). For G149T, the absence of major structural rearrangements is consistent with its near-native redox potential. In G149K, introduction of a positively charged side chain adjacent to Cys30 likely stabilises the catalytic thiolate, accounting for the observed increase in redox potential (Fig. S4).

Despite preservation of oxidising redox potential and loop geometry in both variants, G149T and G149K display reduced catalytic efficiency toward the peptide substrates used in this study. Replacement of Gly149, which lacks a side chain, with either a polar side chain (G149T) or a charged side chain (G149K) introduces additional steric constraints and alters the local chemical environment, potentially affecting short-range steric, hydrogen-bonding, and electrostatic interactions with the peptide substrate. These changes are expected to restrict local conformational sampling and may impair productive substrate engagement and turnover, without disrupting the underlying cis-proline loop architecture.

Together, these structures rationalise the biochemical findings: substitutions at G149 preserve cis-proline loop geometry and redox competence but reduce catalytic efficiency through perturbation of the local active-site environment. In contrast, substitution of P151 disrupts the cis-loop geometry itself, abolishing the hydrogen-bonding platform required for precise cysteine positioning and efficient completion of the catalytic cycle.

To further clarify points 5 and 6 raised by this reviewer, we have also added a paragraph to the Discussion emphasising that substitutions at Gly149 preserve the cis-proline loop geometry but incur a measurable kinetic penalty that reflects perturbation of the local active-site microenvironment. We further highlight that this position likely functions as a chemically tuneable element that modulates substrate engagement and specificity, while the conserved *cis*-proline lock enforces the invariant catalytic geometry. The discussion now includes the following text (lines 377-381, modifications in blue).

Mutational analysis confirmed the pivotal role of this loop. Residue cisP151 is essential for maintaining the cis geometry, and its substitution disrupts the scaffold, reduces the kinetic rate, and prevents complete substrate turnover. In contrast, changes at Gly149 preserve the geometry but alter the local environment, incurring a measurable kinetic penalty in peptide oxidation. This penalty cannot be attributed to altered thermodynamics or disruption of the catalytic scaffold and instead reflects perturbation of the local active-site microenvironment. Accordingly, variation at this position across DsbA homologues is likely to function as a tuning element that modulates substrate specificity rather than general catalytic competence.

We thank both reviewers again for their thoughtful and constructive feedback. We believe that the revisions made in response to these comments have significantly improved the clarity, rigor, and accessibility of the manuscript.
